# Covalent Organic Nanosheets with a Tunable Electronic Structure to Achieve Unprecedented Stability and High‐Performance in Sodium‐Ion Batteries

**DOI:** 10.1002/smll.202502368

**Published:** 2025-04-30

**Authors:** Minseop Lee, Nakyeong Lee, Gumin Kwon, Jae‐Min Oh, Jin Kuen Park, Seung‐Min Paek

**Affiliations:** ^1^ Department of Chemistry Kyungpook National University Daegu 41566 Republic of Korea; ^2^ Department of Chemistry Hankuk University of Foreign Studies Gyenggi‐do Yongin 17035 Republic of Korea; ^3^ Department of Energy and Materials Engineering Dongguk University Seoul 04620 Republic of Korea

**Keywords:** bandgap engineering, covalent organic nanosheets, electron density control, push–pull electronic structure, sodium ion batteries

## Abstract

This study develops a new type of fluorinated covalent organic nanosheets (CONs) as anode materials for sodium‐ion batteries by incorporating an electron‐withdrawing benzothiadiazole (BT) unit and F atom into the framework. These modifications lead to a reduced bandgap and electron density, generating strong permanent dipoles that increased Na^+^ accessible sites within the self‐assembled solid‐state structure. To elucidate the effect of these electronic changes, the Na^+^ storage performance of fluorinated D/A‐CON‐10‐F is compared to that of nonfluorinated D/A‐CON‐10. The reduced electron density in D/A‐CON‐10‐F weakens its interaction with Na^+^, yet enhances ion and charge carrier conductivities, leading to improved electrochemical performance. Notably, D/A‐CON‐10‐F exhibits a reversible discharge capacity of ≈637 mA h g^−1^ at 100 mA g^−1^, maintaining structural stability over 5000 cycles with excellent rate capability. These results demonstrate that dipole engineering in CONs effectively enhances charge transport and long‐term stability, offering a promising strategy for next‐generation sodium‐ion battery anodes.

## Graphical abstract

The new type of covalent organic nanosheets as an anode material for sodium‐ion batteries is developed by the introduction of both the benzothiadiazole motif and fluorine atoms to form the D/A‐CON‐10‐F electrode which finally demonstrates high electrical conductivities and discharge capacity of ≈637 mA h g^−1^ during 500 cycles at the current density of 100 mA g^−1^.

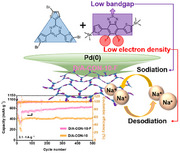

## 1 Introduction

Rechargeable ion batteries are essential for electronic devices—transportation and portable applications.^[^ ^1^ ^]^ Extensive research has explored new secondary batteries, with lithium‐ion batteries (LIBs) gaining attention due to their high energy storage capacity.^[^ ^2^ ^]^ However, due to the limited lithium reserves, large‐scale energy storage is quite challenging. In the carbon neutrality era, the demand for high‐capacity rechargeable batteries for electric vehicles has surged, necessitating cost‐effective, high‐energy‐density alternatives.^[^ ^3^ ^]^ Sodium‐ion batteries (SIBs) are one of the solutions due to the natural abundance of Na, comparable redox potential of Na over Li, and similar intercalation behavior of Na with Li.^[^ ^4^, ^5^ ^]^

While cathode materials in LIBs can be applied to SIBs, conventional LIB anodes are unsuitable for SIBs. Due to larger size of Na^+^ (106 pm) over Li^+^ (76 pm), Na^+^ cannot intercalate efficiently into graphite, limiting discharge capacity. Additionally, high mass of Na impeded the ion transport rate, lowering capability and energy density in SIBs with graphite anodes. Therefore, developing high‐capacity, stable anodes is crucial for SIB commercialization.^[^ ^6^, ^7^ ^]^ Hard carbon has emerged as a promising alternative of graphite in SIBs due to its low cost and electrochemical stability.^[^ ^8^ ^]^ However, it suffers from low energy density and sluggish ion kinetics.^[^ ^9^ ^]^ Thus, novel anode materials are strongly required to enhance reversible capacity, rate performance, and long‐term cycling stability in SIBs.

To address this need, we previously designed highly stable and conductive covalent organic nanosheets (CONs) as polymeric anode materials for sodium‐ion storage.^[^ ^10^ ^]^ The 2D conjugated frameworks, crosslinked with heteroaromatic compounds, exhibit sheet‐like morphologies with intrinsic porosity, which can enhance ionic conductivity.^[^ ^11^ ^]^ Additionally, the delocalized π‐electrons improve electrical conductivity,^[^ ^12^ ^]^ while their rigid networks provide mechanical flexibility, electrolyte insolubility, and thermal and chemical stability.^[^ ^13^ ^]^ The research endeavored on optimizing these structures and identifying key factors affecting SIB performance. Electrical conductivity and electrode polarity, governed by the electronic structure of CON framework, are considered to play crucial roles. Systematic studies revealed that increasing electrical conductivity and sodium ion accessibility enhances SIB performance.^[^ ^10^ ^]^ Conductivity improved owing to planarity, increased S content, and narrowed bandgaps; bandgap reduction was found to be the most significant among them.^[^ ^14^ ^]^ Additionally, fluorination of the framework lowered electron density, enhancing Na^+^ insertion–desertion and improving long‐term stability.^[^ ^15^ ^]^

**Scheme 1:**
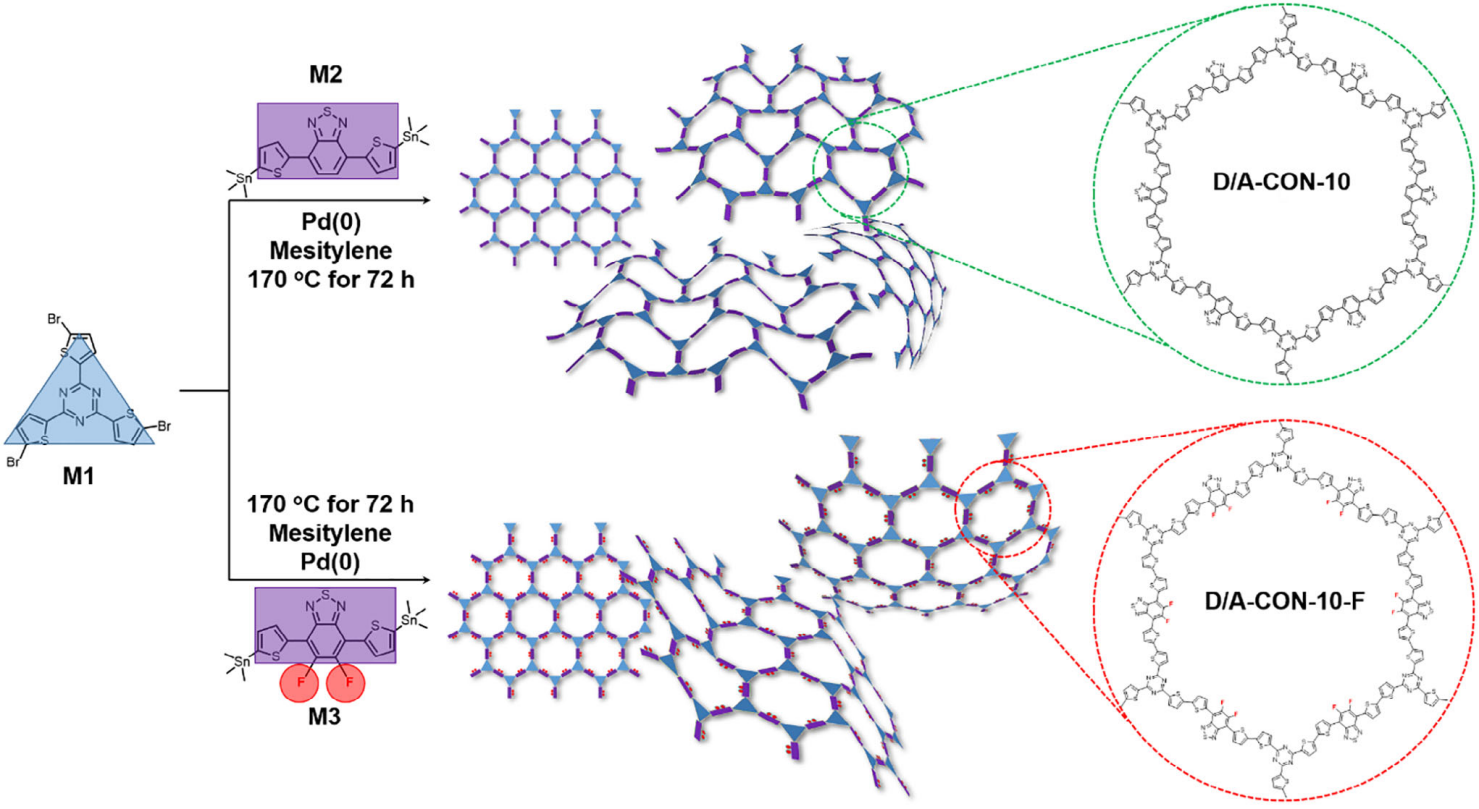
Syntheses and topological and chemical structures of D/A‐CON‐10‐F and D/A‐CON‐10, as well as the chemical structures of their monomers: Pd(0) represents tetrakis (triphenylphosphine)palladium(0), which was used as a catalyst for the Stille cross‐coupling reaction.

This contribution introduces a new type of anode material for high‐performance SIBs by simultaneously narrowing the bandgap and incorporating fluorination. To investigate the combined effect, a series CONs were synthesized via Stille cross‐coupling (**Scheme** **1**): D/A‐CON‐10 (narrow bandgap) and D/A‐CON‐10‐F (fluorinated narrow bandgap). Fluorine incorporation significantly enhanced energy density and electrochemical performance compared to the nonfluorinated counterpart. Notably, D/A‐CON‐10‐F exhibited exceptional stability over 5000 cycles and a high reversible discharge capacity of 637.8 mA h g^−1^ at 100 mA g^−1^, which is not only comparable to its theoretical capacity but also among the best performances reported for organic electrodes. The enhanced performance is attributed to reduced electron density, facilitating Na^+^ insertion–desertion, and a narrowed bandgap, improving charge carrier conductivity. Different from hard carbon, which lacks structural uniformity and tunability,^[^ ^16^, ^17^, ^18^, ^19^ ^]^ CONs can be molecularly tailored in bandgap, heteroatom type, and arrangement,^[^ ^10^, ^11^, ^12^, ^13^, ^14^, ^15^ ^]^ optimizing ion and electron pathways. Consequently, D/A‐CON‐10‐F demonstrates excellent capacity retention, rate capability, and cycling stability, addressing hard carbon's limitations. These findings highlight that synergistic bandgap engineering and fluorination accelerate charge transport and mitigate structural degradation, providing a promising route toward high‐energy‐density SIBs with superior long‐term performance.

## 2 Results and Discussions

### 2.1 Structural and Electronic Characterization

**Figure 1:**
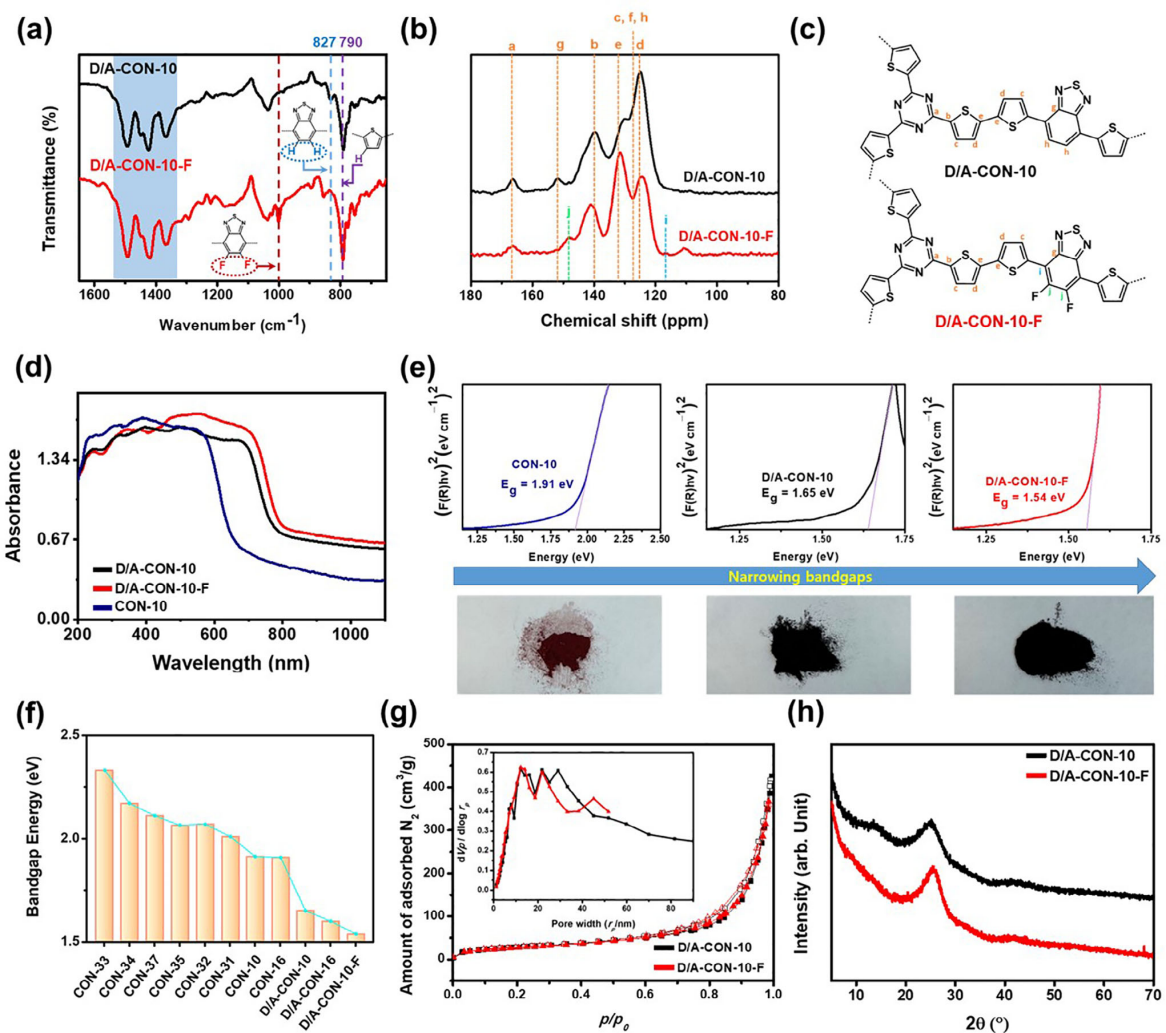
a) Fourier transform infrared (FTIR) spectra of D/A‐CONs with specific bands. b) Solid state cross‐polarization magic angle spinning carbon‐13 nuclear magnetic resonance (CP/MAS ^13^C NMR) spectra of D/A‐CONs, with specific chemical shifts. c) Repeating structures of D/A‐CONs denoting specific carbons matching with NMR spectra. d) Solid state UV–vis absorption spectra of CONs. e) Estimated bandgap (*E*_g_) of CONs from their Tauc plot with the images of powders. f) Comparison in bandgap values of the D/A‐CONs fabricated in this study other CONs in literatures.^[^ ^10^, ^14^, ^15^ ^]^ g) Nitrogen gas adsorption and desorption isotherm profiles (solid: adsorption, empty: desorption; inset: pore size distribution calculated using the Barrett–Joyner–Halenda (BJH) method). h) Powder X‐ray diffraction (PXRD) patterns of D/A‐CONs.

To accurately assess the structural integrity of D/A‐CON‐10 and D/A‐CON‐10‐F (Scheme 1), their Fourier transform infrared (FTIR) spectra were analyzed (**Figure** **1**a). In the fingerprint region (1600–700 cm^−1^), characteristic vibrational peaks corresponding to their building blocks were observed. Both compounds exhibited three distinct vibration peaks of s‐triazine (1500–1300 cm^−1^, shaded blue in Figure 1a),^[^ ^10^, ^11^, ^12^, ^13^, ^14^, ^15^ ^]^ and an out‐of‐plane C_β_ *─*H vibration peak in thiophenes (≈790 cm^−1^). Notably, D/A‐CON‐10 displayed a benzothiadiazole (BT) proton vibration at ≈827 cm^−1^, while D/A‐CON‐10‐F exhibited a distinct C*─*F vibration at ≈1000 cm^−1^,^[^ ^14^, ^20^ ^]^ confirming successful fluorination. These results confirmed the successful synthesis of the CONs as designed (Scheme 1) under the finely‐tuned reaction condition without deterioration of monomers. To investigate the chemical bonding in D/A‐CONs, solid‐state CP/MAS ^13^C NMR analysis was performed (Figure 1b). Both CONs exhibited characteristic C*─*C peaks in aromatic rings; while no signals appeared below 80 ppm, except weak spinning side bands, confirming high purity and no monomer decomposition. The chemical shifts in Figure 1b, highlighted with colored markers, correspond to specific carbons in the repeating structures (Figure 1c). Notably, the electron densities of carbons labeled “i” and “j” (green) were significantly influenced by fluorine incorporation.^[^ ^14^, ^21^ ^]^

The narrowed bandgaps of D/A‐CON‐10 and D/A‐CON‐10‐F were analyzed via solid‐state UV–vis absorption spectra by comparing with previously reported wide‐bandgap CON‐10 (Figure 1d).^[^ ^10^, ^14^, ^15^ ^]^ The absorption onset of D/A‐CONs appeared at higher wavelengths than CON‐10, with D/A‐CON‐10‐F (the most electron deficient unit) showing the largest redshift due to fluorinated BT units forming a strong push–pull electronic structure, effectively narrowing the bandgap.^[^ ^22^, ^23^ ^]^ A narrower bandgap enhances charge carrier mobility and reduces internal resistance, making electron density tunable via fluorinated BT units.^[^ ^14^, ^21^ ^]^ Tauc plot analysis determined bandgap energies of 1.54, 1.65, and 1.91 eV for D/A‐CON‐10‐F, D/A‐CON‐10, and CON‐10, resptectively (Figure 1e). Additionally, the nearly black color of D/A‐CON‐10‐F powder indicated its broad light absorption due to the narrow bandgap.

We have previously reported various CONs to investigate the impact of bandgap on SIB performance.^[^ ^10^, ^11^, ^12^, ^13^, ^14^, ^15^ ^]^ The bandgap comparison of D/A‐CON‐10‐F with other CONs (Figure 1f), revealed that D/A‐CON‐10‐F had significantly lowered bandgap due to its effective push–pull and diode structure, formed by electron‐rich thiophene and electron‐deficient fluorinated BT units. This enhanced diode structure induces strong dipoles in C*─*F bonds, with a negative pole on F atoms and a positive pole on C atoms. As a result, the narrowed bandgap would enhance charge carrier mobility, while strong dipoles lower electron density in the backbone, facilitating sodium ion desorption. These effects are considered to synergistically improve SIB performance by optimizing Na^+^‐ion adsorption–desorption. The N_2_ adsorption/desorption isotherms of D/A‐CON‐10 and D/A‐CON‐10‐F (Figure 1g) exhibit type II behavior by the IUPAC classification,^[^ ^10^, ^24^ ^]^ indicating macroporous or nonporous structures formed by randomly stacked nanosheets.^[^ ^10^, ^15^, ^25^ ^]^ Fluorination has little impact on stacking and macropore formation, resulting in nearly identical isotherm profiles, pore characteristics, and specific surface areas (D/A‐CON‐10: 104.26 m^2^ g^−1^, D/A‐CON‐10‐F: 99.31 m^2^ g^−1^) for both samples. Pore size distribution calculated using the Barrett–Joyner–Halenda (BJH) method indicates that he cumulative pore volume and average pore size of D/A‐CON‐10‐F to be 0.55 m^3^ g^−1^ and 12.2 nm, respectively. The pore sizes of CONs with turbostratic structures range from several nanometers to tens of nanometers over a very wide range, which is due to the multistacked nanosheets consisting of CONs.

As shown in Scheme 1, D/A‐CON‐10 and D/A‐CON‐10‐F form 2D sheets that stack along the crystallographic *c*‐axis in solid state. To analyze this stacking behavior, powder X‐ray diffraction (PXRD) patterns were recorded (Figure 1h). Both CONs exhibit broad halo at ≈2θ = 23°, indicating π–π interactions among aromatic rings, suggesting an amorphous or turbostratic structure similar to reduced graphene oxide (RGO).^[^ ^14^, ^26^ ^]^ The broad PXRD pattern suggests the presence of short‐range π–π stacking; the phenomenon was previous reported in CON systems, where random stacking along the *c*‐axis broadened diffraction peaks.^[^ ^5^, ^10^, ^14^, ^15^ ^]^ Additionally, weak asymmetric shoulders at 2θ = 40°–45° further support turbostratic stacking, common in layered carbonaceous materials like RGO.^[^ ^26^ ^]^ In these structures, misalignment between layers prevents well‐ordered crystalline domains, leading to an overall amorphous or semiamorphous solid‐state organization despite well‐defined 2D backbones of D/A‐CON‐10 and D/A‐CON‐10‐F.

### 2.2 Bandgap Engineering and Morphological Analysis

**Figure 2:**
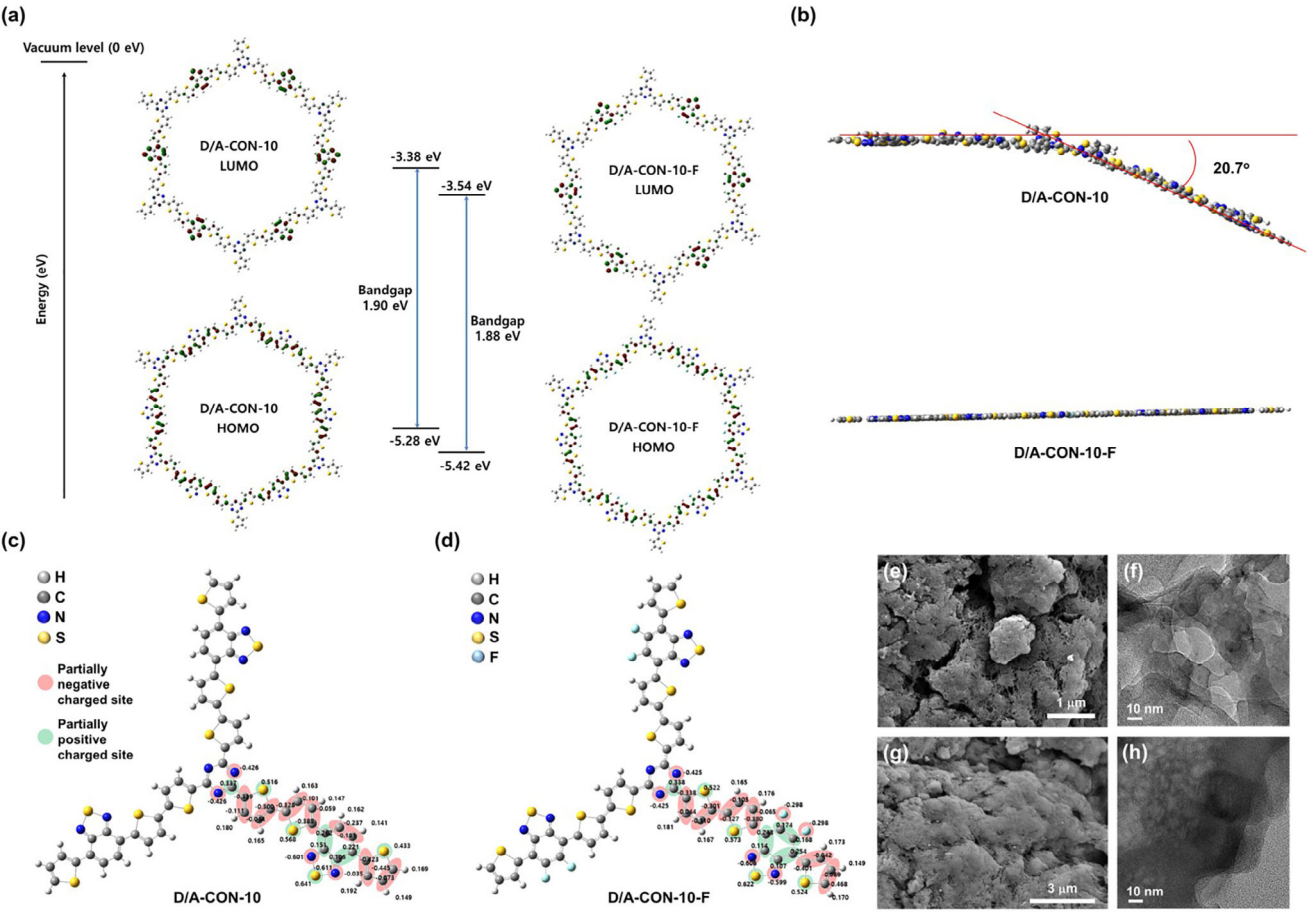
a) Density function theory (DFT) calculation for highlighting the energy levels of the frontier orbitals of D/A‐CON‐10 (left) and D/A‐CON‐10‐F (right) at the B3LPY/6‐31G level. b) Side views of D/A‐CON‐10 (top) and D/A‐CON‐10‐F (bottom) optimized through the DFT calculation at the B3LPY/6‐31G level. Mulliken charge distributions in the unit structures of: c) D/A‐CON‐10 and d) D/A‐CON‐10‐F calculated using DFT at the B3LPY/6‐31G level. Field emission scanning electron microscopy (FE‐SEM) images of: e) D/A‐CON‐10 and g) D/A‐CON‐10‐F. Transmission electron microscopy (TEM) images of f) D/A‐CON‐10 and h) D/A‐CON‐10‐F.

The energy levels of the frontier orbitals—highest occupied molecular orbital (HOMO) and lowest unoccupied molecular orbital (LUMO)—and the planarity of CONs were estimated using density functional theory (DFT) at the B3LYP/6‐31G level (**Figure** **2**a,b). The calculations showed that D/A‐CON‐10‐F had lower HOMO and LUMO levels than D/A‐CON‐10 due to fluorine‐induced orbital stabilization. However, as fluorinated BT contributed more significantly to the LUMO than the HOMO, the LUMO was more stabilized, resulting in a slightly narrower bandgap, consistent with Tauc plot measurements (Figure 1e). Such unequal orbital contribution of BT in the frameworks would plausibly render the more effective diodes required for the push–pull structure.^[^ ^22^, ^23^ ^]^ Furthermore, structural optimization (Figure 2b) revealed that D/A‐CON‐10 had a slight folding angle of 20.7°, whereas D/A‐CON‐10‐F was nearly planar. This increased planarity improves orbital overlap and electron delocalization, making D/A‐CON‐10‐F a more favorable structure for achieving high electrical conductivity and a narrow bandgap.

To analyze the impact of fluorine incorporation in BT units, Mulliken charges of D/A‐CONs were estimated using DFT at the B3LYP/6‐31G level (Figure 2c,d). The backbone of D/A‐CON‐10 had negatively charged nitrogen, thiophene carbon, and BT hydrocarbon atoms (shaded area with pink regions in Figure 2c), while s‐triazine carbon, thiophene sulfur, and some BT carbon atoms carried positive charges (blue‐shaded areas in Figure 2c). Hydrogen atoms exhibited positive charges throughout. However, in D/A‐CON‐10‐F, fluorine bonding induced more positive sites on BT carbon atoms (Figure 2d). Since Na^+^‐ions interact with π‐conjugated backbones in SIBs, the fluorination‐driven reduction in electron density in D/A‐CON‐10‐F would facilitate desodiation,^[^ ^15^ ^]^ resulting in improving SIB performance.

The solid‐state morphologies of D/A‐CON‐10 and D/A‐CON‐10‐F were analyzed using FE‐SEM (Figure 2e,g), to analyze the self‐assembled structure. Both frameworks exhibited randomly agglomerated structures, consistent with PXRD results. The D/A‐CON‐10‐F displayed small globular features; on the other hand, D/A‐CONs exhibited somewhat clumped sheet‐like features, possibly due to the different planarity degrees in both samples. To further examine intracrystalline structures, transmission electron microscopy (TEM) imaging was conducted (Figure 2f,h). The results revealed the random stacking of various sizes of CONs in a turbostratic manner. Nonetheless, some fringed lines were observed at the edge of particles, implying nanosheet‐like features. This confirmed the successful fabrication of a series of D/A‐CONs using the process illustrated in Scheme 1.

To gain further insights in nanosized crystalline domain of D/A‐CON‐10‐F stacked via π–π interactions, HR‐TEM images (Figure S1a, Supporting Information) were obtained. A selected area (blue dotted rectangle) exhibited lattice fringes, and the fast Fourier transform (FFT) pattern (inset in Figure S1b, Supporting Information) revealed a *d*‐spacing of ≈0.34 nm, corresponding to the (002) plane of D/A‐CON‐10‐F (the inset image in Figure S1b, Supporting Information). To further examine lattice fringes, an inverse FFT image (Figure S1b, Supporting Information) was obtained, showing a polycrystalline domain with randomly arranged fringes. Brightness profile analysis (Figure S1c, Supporting Information) confirmed periodic bright signal maxima with peak‐to‐peak distances of 0.33–0.35 nm, consistent with π–π stacking in crystalline phases. These findings suggested the formation of a well‐aligned 2D structure by the D/A‐CON‐10‐F, which could play a critical role in its charge carrier transport pathways. Similar results were observed in other crystalline domains (Figure S1d–h, Supporting Information), confirming a well‐aligned 2D structure crucial for charge carrier transport. The energy dispersive X‐ray spectroscopy (EDS) elemental distribution maps for D/A‐CON‐10‐F and D/A‐CON‐10 are presented in Figure S2 (Supporting Information).

### 2.3 Chemical Composition and Bonding Characteristics

**Figure 3:**
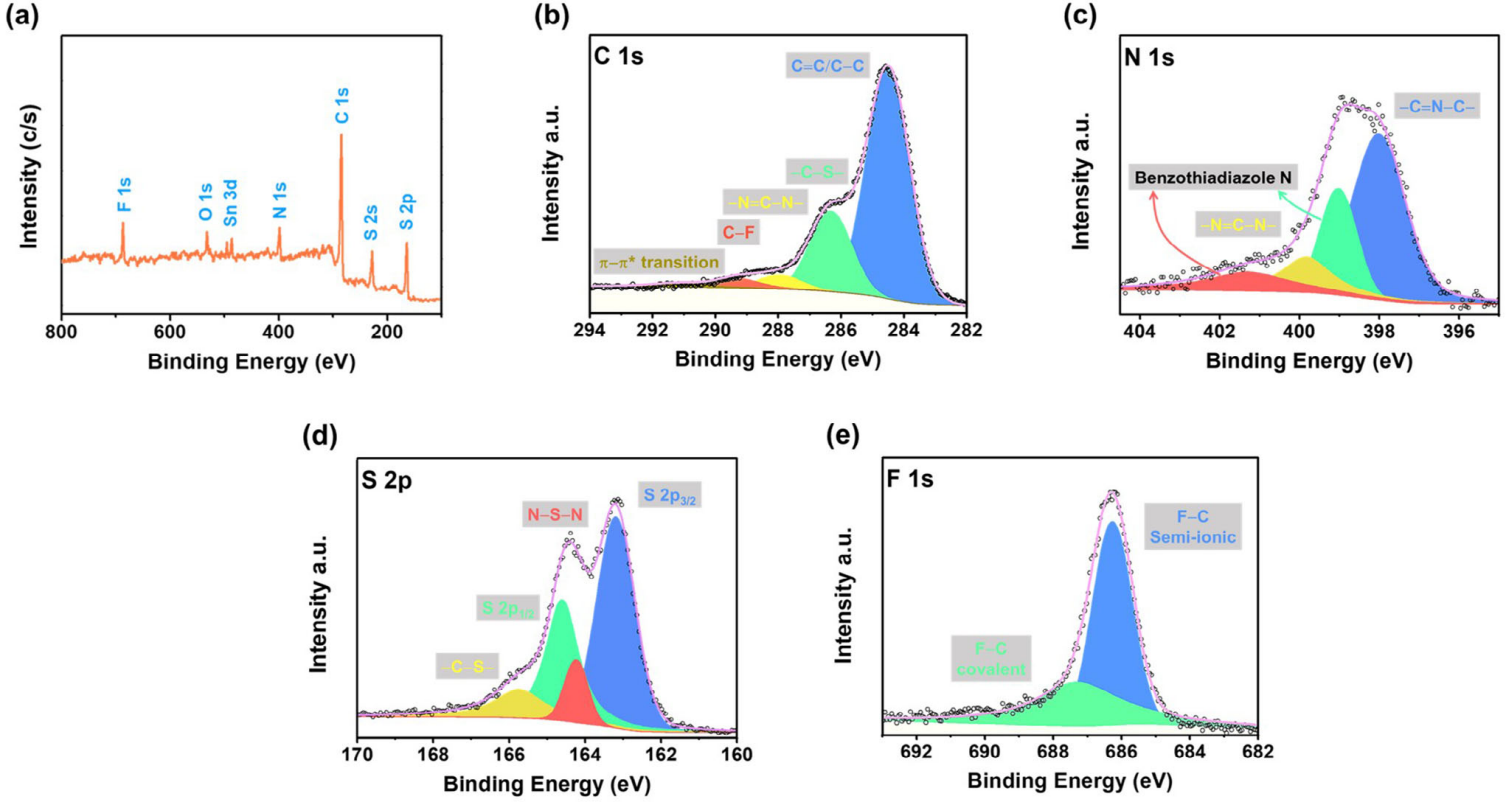
a) X‐ray photoelectron spectroscopy (XPS) survey spectra of D/A‐CON‐10‐F and high‐resolution XPS profiles at peaks of b) C 1s, c) N 1s, d) S 2p, and e) F 1s.

The chemical composition and bonding nature of D/A‐CON‐10‐F were analyzed using X‐ray photoelectron spectroscopy (XPS) (**Figure** **3**a–e; and Figure S3, Supporting Information). The survey spectrum confirmed the presence of key elements, such as S, C, N, and F and the absence of any discernible Pd (Figure 3a). Relative atomic percentages (Table S1, Supporting Information) showed C and S as the most abundant elements, with the other significant elements like N and F in the framework. The C 1s spectrum exhibited peaks at 284.4, 286.2, 288.0, 289.2, and 290.9 eV, corresponding to C═C, C*─*S, N═ C*─*N, C*─*F bonds, and π–π* transitions, respectively (Figure 3b).^[^ ^6^, ^15^ ^]^ The N 1s spectrum displayed peaks at 398.0 and 399.8 eV for triazine units and 399.1 and 401.2 eV for BT units (Figure 3c).^[^ ^27^ ^]^ Peaks for S 2p_3/2_ and S 2p_1/2_ appeared at 163.2 and 164.6 eV, assigned to C*─*S*─*(C) and N*─*S*─*(N) bonds, while an additional peak at ≈164.2 eV was attributed to N*─*S bonds in BT units (Figure 3d).^[^ ^14^, ^27^ ^]^

The F 1s spectrum was deconvoluted into two peaks at 686.2 and 687.3 eV, indicating semi‐ionic and covalent bonding (Figure 3e).^[^ ^15^, ^28^ ^]^ The Sn and O peaks were detected at low intensities, confirming minimal SnO_2_ content in CONs (Figure S3, Supporting Information). Analysis of full width at half maximum (FWHM) and peak areas (Table S2, Supporting Information) corroborated that the CON frameworks were robustly synthesized without any signs of chemical deterioration (Scheme 1, Supporting Information).

### 2.4 Electrochemical Performance and Na^+^ Storage Mechanism

**Figure 4:**
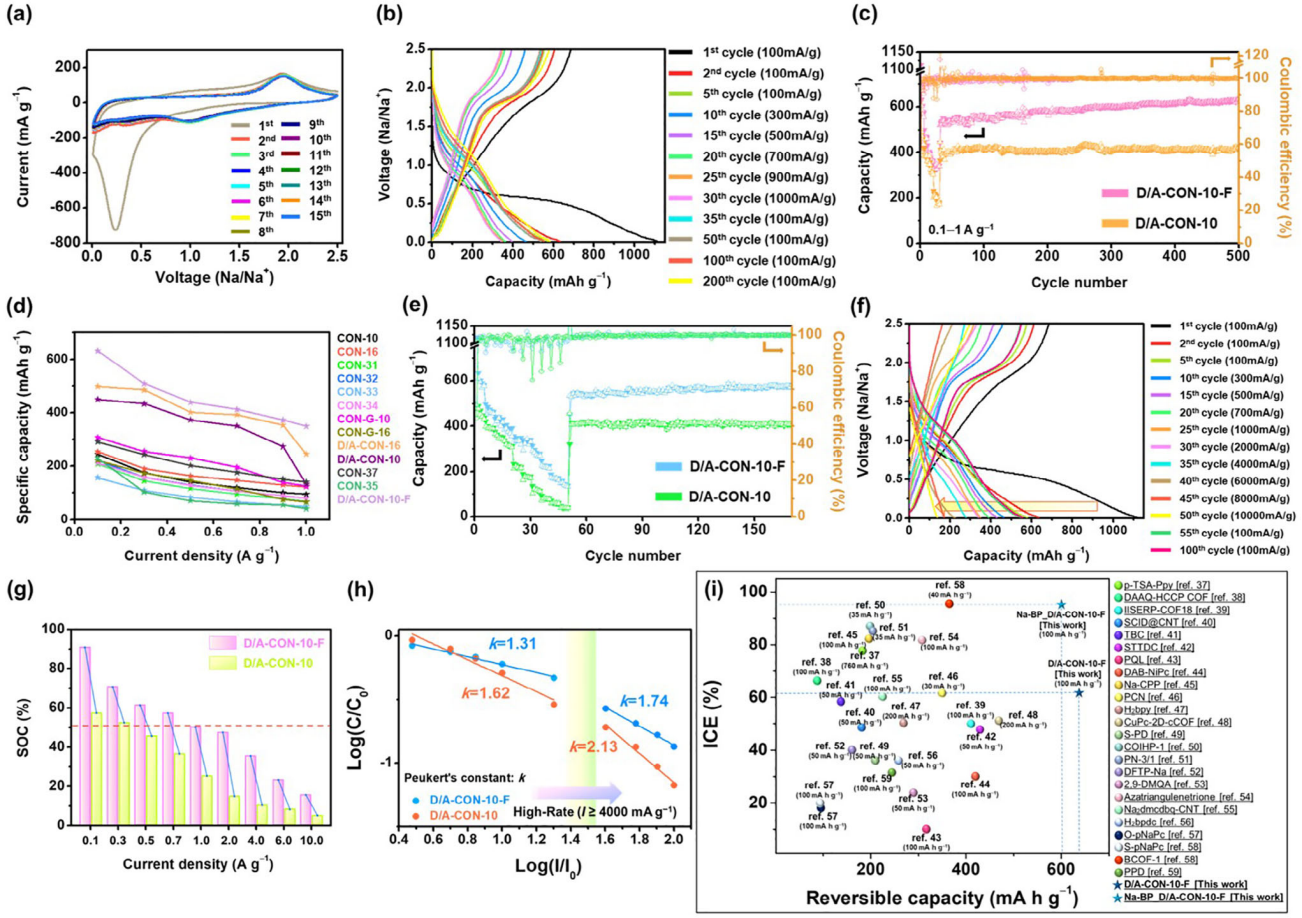
a) Cyclic voltammetry (CV) profiles of D/A‐CON‐10‐F at a scan rate of 0.1 mV s^−1^. b) Galvanostatic charge–discharge (GCD) profiles of D/A‐CON‐10‐F electrode at different current densities (100–1000 mA g^−1^). c) Rate performance of D/A‐CON‐10‐F and D/A‐CON‐10 electrodes measured at different current densities (100–1000 mA g^−1^). d) Comparison of the rate performance of the D/A‐CON‐10‐F electrode with other CON‐based electrodes.^[^ ^5^, ^10^, ^14^, ^15^ ^]^ e) Rate performance of D/A‐CON‐10‐F and D/A‐CON‐10 electrodes measured at different current densities (100–10 000 mA g^−1^). f) Charge–discharge profiles of D/A‐CON‐10‐F electrodes at different current densities (100–10000 mA g^−1^). g) Comparison of the state of charge (SOC) of D/A‐CON‐10‐F (1 C = 704.24 mA h g^−1^) and D/A‐CON‐10 (1 C = 745.52 mA h g^−1^) electrodes at the galvanostatic stage at various current densities. h) Determination of the Peukert's constant of the D/A‐CON‐10‐F and D/A‐CON‐10 electrodes from the slope. i) Comparison of reversible capacity and ICE values of recently reported organic‐based anodes for SIBs.^[^ ^37^, ^38^, ^39^, ^40^, ^41^, ^42^, ^43^, ^44^, ^45^, ^46^, ^47^, ^48^, ^49^, ^50^, ^51^, ^52^, ^53^, ^54^, ^55^, ^56^, ^57^, ^58^, ^59^ ^]^

Cyclic voltammetry (CV) of the D/A‐CON‐10‐F electrode was measured from 0.01 to 2.5 V at 0.1 mV s^−1^ scan rate over initial 15 cycles (**Figure** **4**a). In the 1st cycle, a large irreversible reduction peak at ≈0.25 V was observed by the formation of solid electrolyte interphases (SEI), but CV curves were stabilized with repeated cycling. Moreover, the negligible decrease in the current response during cycles indicated high cycle stability. In the CV curves of D/A‐CON‐10‐F electrode, redox peaks were identified at ≈0.99 and 1.93 V (Figure 4a), attributable to interactions between heteroatoms (e.g., N, S) within the D/A‐CON‐10‐F framework and Na^+^.^[^ ^14^, ^15^ ^]^ Notably, the redox peaks of D/A‐CON‐10‐F with reduced voltage gap compared to previous D/A‐CON‐10 indicated easy insertion–desertion of Na^+^ into this framework.^[^ ^14^ ^]^ The narrow voltage difference suggests reduced polarization, implying insertion–desertion with lower energy barriers during electrochemical reactions. It was noteworthy that the D/A‐CON‐10‐F electrode does not exhibit any characteristic redox peaks of SnO_2_, that the trace amount (3.7 wt%) of SnO_2_ in the electrode has little effect on the overall electrochemical process.^[^ ^29^ ^]^ In addition, the inductively coupled plasma (ICP) result with 4.1 wt% of Sn was well consistent with the XPS elemental analysis (Table S1, Supporting Information).

The CV peaks observed in the D/A‐CON‐10‐F electrode are relatively broad rather than sharp and distinct, indicating that the interactions between Na^+^ and the electrode framework are primarily due to relatively weak noncovalent bonding.^[^ ^14^, ^15^ ^]^ These results indicate that Na^+^ storage in the D/A‐CON‐10‐F electrode occurs not only at specific redox‐active sites but is also distributed evenly throughout the framework, a characteristic behavior of pseudocapacitive properties.^[^ ^14^, ^15^ ^]^

The galvanostatic charge–discharge (GCD) profiles of the D/A‐CON‐10‐F electrode at current densities ranging from 100 to 1000 mA g^−1^ (Figure 4b) were compared with those of the D/A‐CON‐10 electrode (Figure S4, Supporting Information). The 1st discharge/charge capacities and initial Coulombic efficiency (ICE) of D/A‐CON‐10‐F were 1112.3/684.8 mA h g^−1^ and 61.6%, respectively. The ICE of the D/A‐CON‐10‐F electrode was significantly higher than those of previously reported CON‐based electrodes (Figure S5, Supporting Information).^[^ ^10^, ^14^, ^15^ ^]^ It was attributed to the lower bandgap of D/A‐CON‐10‐F than other CONs, enhancing electron mobility. The enhanced electron mobility effectively reduced electron transfer resistance during the initial charge–discharge process, improving the ICE of the electrode.

From the 2nd cycle onward, the CV curves and charge/discharge profiles of D/A‐CON‐10‐F overlap almost entirely, maintaining a stable Coulombic efficiency of ≈100%, indicating excellent reversibility. However, for practical SIB applications, optimizing the ICE to 100% is essential, typically requiring a presodiation process.^[^ ^30^ ^]^ As D/A‐CON‐10‐F has a higher redox potential than Na‐biphenyl (Na‐BP, 0.12 V vs Na/Na^+^),^[^ ^31^ ^]^ chemical presodiation can be applied during the electrode fabrication stage. In this study, chemical presodiation was performed by immersing the electrode in a Na‐BP solution for 1 min (Figure S6a, Supporting Information). This treatment significantly increased the ICE to ≈95% (Figure S6b, Supporting Information), demonstrating that a simple chemical process can effectively enhance initial charge–discharge efficiency.

The rate capacities at 100 (2th cycle), 300 (10th cycle), 500 (15th cycle), 700 (20th cycle), 900 (25th cycle), and 1000 (30th cycle) mA g^−1^ were 631.1, 469.3, 399.6, 362.7, 349.5, and 342.5 mA h g^−1^, respectively. Furthermore, after returning to a current density of 100 mA g^−1^, a reversible capacity of 637.8 mA h g^−1^ was observed at the 500th cycle. This confirms that both the push–pull structure and F‐functional groups in D/A‐CON‐10‐F synergistically reduced the bandgap and established a strong permanent dipole, significantly enhancing the mobility of electrons and ions and substantially improving the Na^+^ storage capacity.

Figure S7a,b (Supporting Information) presents the d*Q*/d*V* curves of D/A‐CON‐10‐F and D/A‐CON‐10, derived from their GCD profiles at various current densities. As current density increased, both electrodes showed decreased peak positions and intensities in their d*Q*/d*V* curves. The variations in the peak positions of d*Q*/d*V* profiles (D/A‐CON‐10‐F and D/A‐CON‐10 electrodes) indicates that the polarization tends to increase as the current density increases (Figure S7c, Supporting Information). However, the peak positions of the d*Q*/d*V* of the D/A‐CON‐10‐F electrode were less varied, and the peak intensities were higher than those of D/A‐CON‐10 electrode, suggesting the less polarization and charge resistance of D/A‐CON‐10‐F electrode. This also indicated that the D/A‐CON‐10‐F electrode, consisting of both push–pull and strong dipole moieties, could facilitate faster electron transport even at high current densities.^[^ ^15^, ^32^ ^]^ Thus, D/A‐CON‐10‐F electrode showed improved rate performance with high charge–discharge efficiency and stable capacity retention across different current densities.

Figure 4c shows the rate performance of D/A‐CON‐10‐F and D/A‐CON‐10 from 100 to 1000 mA g^−1^. The D/A‐CON‐10‐F electrode retained full capacity after 500 cycles when the current density was reverted to 100 mA g^−1^ after the 30th cycle, indicating minimal irreversible capacity loss. This stability contrasts with typical organic electrodes, which suffer from side reactions, solubility issues, and slow Na^+^ kinetics.^[^ ^33^ ^]^ The slight capacity increase during cycling suggests progressive ion channel opening, enhancing ion transport and long‐term stability. When the current density returned to 100 mA g^−1^ after 30 cycles, the D/A‐CON‐10‐F electrode achieved a maximum reversible capacity of 640 mA h g^−1^, surpassing previously reported CON‐based electrodes (Figure 4d).^[^ ^5^, ^10^, ^14^, ^15^ ^]^ This highlights its exceptional resilience under varying charge–discharge conditions.

The high‐rate performance of D/A‐CON‐10‐F and D/A‐CON‐10 was compared at current densities up to 10 000 mA g^−1^ (Figure 4e). D/A‐CON‐10‐F exhibited superior capacity retention, recovering close to its maximum capacity when the current density returned to 100 mA g^−1^ after 50 cycles at 10 000 mA g^−1^, demonstrating stability under ultrafast charge–discharge conditions. D/A‐CON‐10‐F achieved high specific capacities of 247, 253, 186, and 157 mA h g^−1^ at 2000, 4000, 6000, and 10 000 mA g^−1^, respectively (Figure 4f), outperforming D/A‐CON‐10. The D/A‐CON‐10‐F electrode also exhibited a gentle discharge slope, indicating significant charge storage around 1 V. This suggests a pseudocapacitive mechanism, where charge accumulates through surface reactions and ion insertion without distinct phase transitions.^[^ ^6^, ^34^ ^]^ This mechanism minimizes structural deformation and enables rapid ion and electron transport.^[^ ^6^, ^34^ ^]^

The high stability and excellent high‐rate performance of D/A‐CON‐10‐F make it suitable for long‐term cycling in high‐power applications. Specifically, at 100 mA g^−1^, its reversible discharge capacity below 1 V exceeds 475.3 mA h g^−1^ (≈75% of total capacity). Even at 8000 mA g^−1^, this fraction remains ≈72%, indicating stable charge transport and Na^+^ insertion–desertion under high‐power conditions. During galvanostatic charging, the state of charge (SOC) of D/A‐CON‐10‐F reached ≈50.5% as current increased from 100 (≈0.14 C) to 1000 mA g^−1^ (≈1.42 C), significantly outperforming D/A‐CON‐10 (≈25.4%) under the same conditions (Figure 4g).^[^ ^35^ ^]^ According to the Na⁺ binding model and theoretical capacity calculations (Figure S8, Supporting Information), theoretical capacity of D/A‐CON‐10‐F (704.2 mA h g^−1^) closely matches its discharge capacity of 637 mA h g^−1^ after 500 cycles at 100 mA g^−1^. This confirms that optimizing electron density and bandgap in D/A‐CON‐10‐F is an effective strategy for developing ultrahigh‐performance SIB electrodes.

Figure 4h compares the Peukert constants (*k*) of D/A‐CON‐10‐F and D/A‐CON‐10 electrodes at varying current densities. The Peukert effect describes how increasing charge/discharge rates lead to higher reaction polarization and reduced electrochemical performance (see the Supporting Information).^[^ ^36^ ^]^ A *k*‐value near 1 indicates minimal performance loss with increasing charge rates, while higher values suggest greater polarization and capacity loss. The *k*‐value of D/A‐CON‐10‐F more effectively remained closer to 1 than D/A‐CON‐10, especially at current densities above 4000 mA g^−1^, demonstrating stable performance even at high current densities. This stability is attributed to the push–pull structure and F‐functional groups, which enhance electron/ion mobility and reduce resistance, lowering reaction polarization.

Figure 4i illustrates the comparative results of reversible capacity and ICE of organic‐based anodes reported in recent literature, with detailed information summarized in Table S3 (Supporting Information).^[^ ^37^, ^38^, ^39^, ^40^, ^41^, ^42^, ^43^, ^44^, ^45^, ^46^, ^47^, ^48^, ^49^, ^50^, ^51^, ^52^, ^53^, ^54^, ^55^, ^56^, ^57^, ^58^, ^59^ ^]^ The D/A‐CON‐10‐F electrode demonstrated outstanding reversibility, improved ICE, and excellent long‐term cycling stability compared to other organic‐based anodes (Figure 4i).

**Figure 5:**
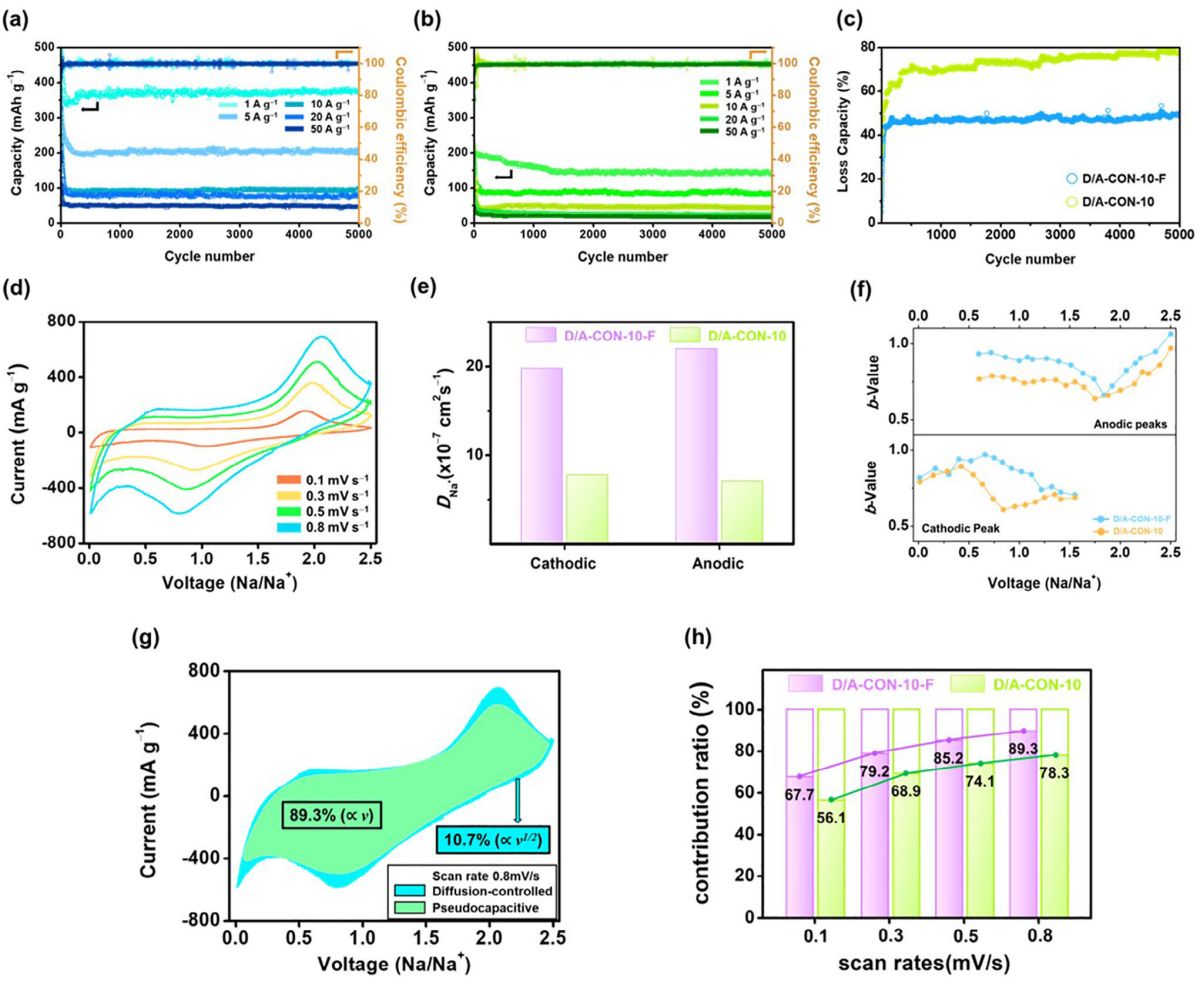
a) Long‐term cycling performance and Coulombic efficiency (CE) of the a) D/A‐CON‐10‐F and b) D/A‐CON‐10 electrodes at various current densities (1, 2, 10, 20, 50 A g^−1^). c) Capacity loss of D/A‐CON‐10‐F and D/A‐CON‐10 electrodes at 50 A g^−1^. d) CV profiles of D/A‐CON‐10‐F electrode at various scan rates (0.1–0.8 mV s^−1^). e) Na^+^‐ion diffusion coefficient ($D_{Na^{+}}$) calculated using the Randles–Sevcik equation. f) Comparison of the *b*‐values of the samples at different voltage (0.01–2.5 V). g) Separation of the pseudocapacitive and diffusion‐controlled charge storage processes at the D/A‐CON‐10‐F electrode. h) Comparison of the pseudocapacitive contributions of the D/A‐CON‐10‐F and D/A‐CON‐10 electrodes.

**Figure** **5**a,b shows the long‐term cycling performance of D/A‐CON‐10‐F and D/A‐CON‐10 over 5000 cycles at high current densities (1–50 A g^−1^). Without precycling, D/A‐CON‐10‐F maintained 375 mA h g^−1^ at 1 A g^−1^, significantly outperforming D/A‐CON‐10 (142 mA h g^−1^). Even at 50 A g^−1^, D/A‐CON‐10‐F retained 46 mA h g^−1^ with an average Coulombic efficiency of ≈99.9%, demonstrating excellent stability at ultrahigh current densities. Compared to previously reported organic electrodes (Table S3, Supporting Information), it exhibited superior reversible capacity and cycling stability. Figure 5c compares capacity degradation over 5000 cycles at 50 A g^−1^. While D/A‐CON‐10‐F showed an initial performance drop, it stabilized after ≈100 cycles. In contrast, D/A‐CON‐10 experienced continuous capacity loss, indicating the superior structural stability of D/A‐CON‐10‐F for long‐term Na^+^ storage.

Ex situ FTIR measurements were conducted to assess the reversible structural changes of D/A‐CON‐10‐F during cycling (Figure S9, Supporting Information). The FTIR spectrum, measured without electrolyte contact, showed characteristic peaks for C═C bonds and the triazine moiety. After electrolyte contact, SEI formation was observed, indicated by PF_6_^−^ adsorption. During the 1st discharge, Na^+^ insertion reduced the intensity of C═C and triazine peaks, but these were restored after charging, confirming structural reversibility. Even after 300 cycles, the D/A‐CON‐10‐F framework retained its chemical bonds, demonstrating minimal degradation during long‐term cycling.^[^ ^6^, ^60^ ^]^

Structural and elemental changes in the D/A‐CON‐10‐F electrode after cycling were examined using HR‐TEM and EDS (Figure S10, Supporting Information). Even after 300 cycles, the layered nanosheet structure remained intact, demonstrating resilience against Na^+^ insertion–desertion while maintaining smooth ion diffusion. EDS analysis confirmed the homogeneous distribution of C, N, S, and F within the electrode.

To investigate Na^+^ storage kinetics, CV curves were recorded at scan rates from 0.1 to 0.8 mV s^−1^ (Figure 5d). In Figure 5e, the Na⁺ diffusion coefficient ($D_{Na^{+}}$) was calculated using the Randles–Sevcik equation (see the Supporting Information).^[^ ^61^ ^]^ The D/A‐CON‐10‐F electrode showed higher Na⁺ diffusion coefficients (*D*_Na+_) at both cathodic and anodic peaks than D/A‐CON‐10, indicating improved ionic conductivity and smoother Na^+^ transport. In Figure 5f, the *b*‐value, determined from the slope of the log i versus log *v* graph, indicates the Na⁺ storage process type (see the Supporting Information).^[^ ^62^ ^]^ A *b*‐value of 0.5 indicates a diffusion‐controlled Faradaic process, while a value near 1 suggests pseudocapacitive behavior. The D/A‐CON‐10‐F electrode exhibited a *b*‐value close to 1 across the measured voltage range, confirming that Na^+^ storage was primarily governed by a surface charge storage mechanism. Additionally, its *b*‐value was generally higher than that of D/A‐CON‐10, indicating superior electron mobility for Na^+^ accommodation. For a quantitative comparison, the total stored charge at each scan rate was divided into diffusion‐independent pseudocapacitive (*k*_1_ *v*) and diffusion‐controlled ($k_{2}v^{1/2}$) contributions using the method proposed by Dunn et al. (see the Supporting Information).^[^ ^62^ ^]^

Figure 5g shows the pseudocapacitive contribution for D/A‐CON‐10‐F at 0.8 mV s^−1^, while Figure 5h compares storage contributions at various scan rates. For D/A‐CON‐10‐F, the pseudocapacitive contribution was 67.7%, 79.2%, 85.2%, and 89.3% at 0.1, 0.3, 0.5, and 0.8 mV s^−1^, respectively—higher than D/A‐CON‐10 and among the highest reported for CON‐based electrodes.^[^ ^5^, ^10^, ^14^, ^15^ ^]^

### 2.5 Charge Transport Kinetics and Interfacial Resistance Analysis

**Figure 6:**
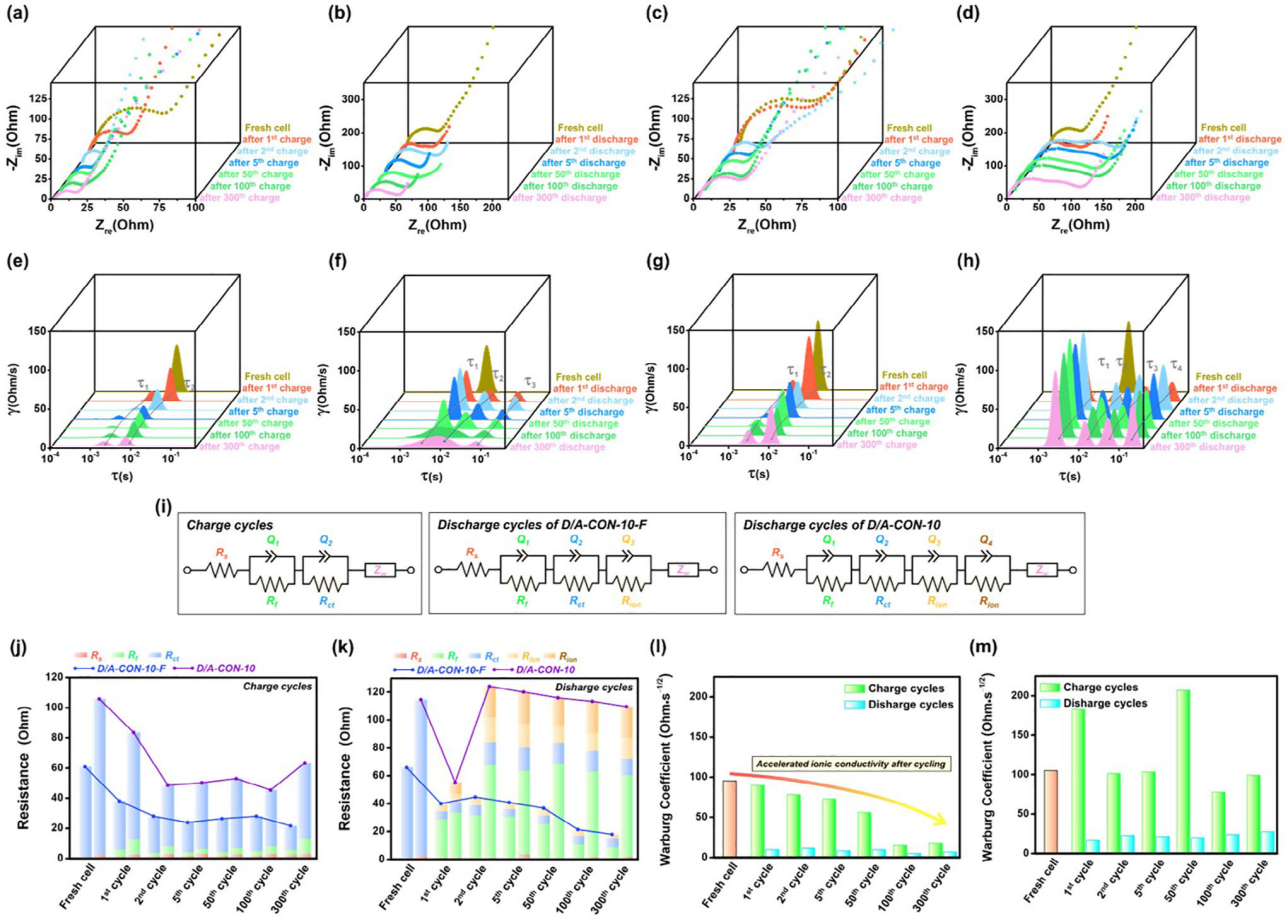
a) Nyquist plot of the a,b) D/A‐CON‐10‐F and c,d) D/A‐CON‐10 electrodes measured up to the 300th cycle. Distribution of relaxation time (DRT) plot of the e,f) D/A‐CON‐10‐F and g,h) D/A‐CON‐10 electrodes. i) equivalent circuit model (ECM) fitting the electrochemical impedance spectroscopy (EIS) data of D/A‐CON‐10‐F and D/A‐CON‐10 electrodes before and after the cycle. Comparison of the ECM fitting parameters after j) charge and k) discharge cycles. Warburg coefficients of the l) D/A‐CON‐10‐F and m) D/A‐CON‐10 electrodes.

Nyquist plots of D/A‐CON‐10‐F (**Figure** **6**a,b) and D/A‐CON‐10 (Figure 6c,d) after various cycles, showed semicircles in high/mid‐frequency regions and a diffusion line in the low‐frequency region, characterizing that ion resistance was prominent in the mid‐frequency region during discharge, reflecting internal resistance changes during cycling. Phase changes in resistance were analyzed using distribution of relaxation time (DRT) plots (Figure 6e–h) and the Bode plot (Figure S11, Supporting Information).^[^ ^63^ ^]^ The DRT plots showed two peaks: τ_1_ (SEI layer impedance) and τ_2_ (charge transfer impedance). The interfacial resistance decreased with cycling, indicating electrode activation. Notably, *τ*_1_ and τ_3_ decreased more significantly for D/A‐CON‐10‐F, suggesting a more efficient electron conduction pathway. After 100 cycles, the internal resistance of D/A‐CON‐10‐F decreased faster than that of D/A‐CON‐10, highlighting its superior interfacial optimization.

The DRT plots revealed additional resistance elements (τ_3_, τ_4_) in the mid‐frequency region after discharge, indicating structural changes due to Na^+^ insertion (Figure 6f,h). These ion resistances disappeared after charging, confirming reversible structural changes. However, D/A‐CON‐10‐F showed a greater decrease in τ_3_, suggesting superior stability for reversible Na^+^ storage. In contrast, D/A‐CON‐10 exhibited higher resistance immediately after sodiation, highlighting the structural advantages of D/A‐CON‐10‐F in reducing internal resistance and facilitating charge transport. Internal resistance contributions were analyzed using equivalent circuit models (ECMs) (Figure 6i). Resistance components included *R*_s_ (ohmic resistance), *R*_f_ (SEI layer resistance), *R*_ct_ (charge transfer resistance), and *R*_ion_ (ionic resistance).^[^ ^15^, ^64^ ^]^ Among these, *R*_ct_ was the dominant resistance during charging, with D/A‐CON‐10‐F exhibiting a lower *R*_ct_ (17 Ω at the 300th cycle) compared to D/A‐CON‐10 (40 Ω), indicating superior conductivity due to its narrower bandgap (Figure 6j). Figure 6k shows that the internal resistance of D/A‐CON‐10 was five times higher than D/A‐CON‐10‐F after 300 cycles, attributed to a rapid increase in *R*_ion_ after the first discharge. Warburg coefficient analysis (Figure 6l,m) confirmed that D/A‐CON‐10‐F exhibited superior Na⁺ mobility and ionic conductivity, demonstrating that bandgap narrowing and fluorination enhance Na^+^ insertion–desertion and ion storage kinetics in organic electrodes.^[^ ^65^ ^]^

## 3 Conclusion

This study successfully synthesized D/A‐CON‐10‐F as an advanced anode material by integrating bandgap narrowing and fluorination for high‐performance SIBs. The incorporation of BT units reduced the bandgap and electron density, while fluorine further amplified these effects. As a result, D/A‐CON‐10‐F exhibited lower charge resistance and weaker Na⁺ binding affinity, enhancing electron transfer and electrochemical performance. With these design features, D/A‐CON‐10‐F achieved superior ICE, minimized side reactions, excellent capacity retention under ultrafast cycling, and improved Na^+^ storage kinetics. It maintained a reversible discharge capacity of ≈637 mA h g^−1^ over 500 cycles at 100 mA g^−1^ without structural deterioration. The push–pull structural design, with fluorinated BT moieties, reduced electron density, enhancing charge carrier mobility, and ion diffusion. These synergistic effects accelerated reversible Na^+^ storage, establishing D/A‐CON‐10‐F as a promising next‐generation anode for high‐performance SIBs.

## 4 Experimental Section

### Materials

2,4,6‐tris(5‐bromothiophene‐2‐yl)‐1,3,5‐triazine, (trimethylstannyl thiophen‐2‐yl)benzo[c]^[^ ^1^, ^2^, ^5^ ^]^‐thiadiazole, and 5,6‐difluoro‐4,7‐bis(5‐(trimethylstannyl)thiophene‐2‐yl)benzo[c][1,2,5]thiadiazole were purchased from Luminescence Technology. Tetrakis(triphenylphoshine)palladium(0), and mesitylene were purchased from Sigma‐Aldrich. All reagents are stored in a glove box charged with N_2_ gas.

### Characterization

PXRD measurements were carried out using an Empyrean Series 2 diffractometer (PANalytical B.V., The Netherlands) equipped with Ni‐filtered Cu‐Kα radiation (*λ* = 1.5406 Å). A 1.60‐mm antiscatter slit and a 5‐mm fixed incident beam mask were applied. The diffraction patterns were collected over a range of 3°–70° with a step size of 0.0525° and a scan rate of 0.75 s per step. The N_2_ adsorption/desorption isotherms were obtained using a Belsorp‐mini II analyzer (Bel Japan Inc.). FTIR spectra were acquired using a Jasco FT/IR‐4100 spectrometer, covering the wavenumber range of 650–4000 cm^−1^. SEM images were captured using a Quanta 250 FEG system (FEI Company, Hillsboro, OR). For TEM analysis, the samples were ultrasonically treated in ethanol solvent for 30 min and then drop‐cast onto lacey carbon film/200 mesh Cu grids (EMS). The FTIR spectra of the products were collected using the KBr pellet method on a Nicolet iS5 FT‐IR spectrometer (Thermo Fisher Scientific Inc., USA). The UV–vis absorption spectra of the products were recorded on an EVOLUTION 220 UV–vis spectrophotometer (Thermo Fisher Scientific Inc., USA) using BaSO_4_ as a reference. A K‐Alpha XPS system (Thermo Fisher Scientific Inc., USA) characterized the elemental composition and surface state. The CP/MAS ^13^C NMR data were collected on a 500 MHz spectrometer (AVANCE III HD, Bruker, Germany) at the KBSI Western Seoul Center, using an HX CPMAS probe with a 4 mm o.d. zirconia rotor.

### Synthesis of D/A‐CON‐10‐F

2,4,6‐tris(5‐bromothiophene‐2‐yl)‐1,3,5‐triazine(0.05 g, 8.863 × 10^−5^ mol; Luminescence Technology), 5,6‐difluoro‐4,7‐bis(5‐(trimethylstannyl)thiophene‐2‐yl)benzo[c][1,2,5]thiadiazole (0.0877 g, 1.33 × 10^−4^ mol; Luminescence Technology), tetrakis(triphenylphosphine)palladium(0) (0.0041 g, 4 mol%; Alfa Aesar), and mesitylene (3 mL; Sigma‐Aldrich), all stored in a glove box charged with N_2_ gas, was refluxed at 170 °C for 72 h upon stirring and poured into methanol. The produced black precipitate was collected by filtration, purified by sequential Soxhlet extraction with methanol, ethanol, dichloromethane, tetrahydrofuran, methanol, ethanol, and acetone (4 h per extraction step), and finally dried in a vacuum for 12 h.^[^ ^14^ ^]^

### Synthesis of D/A‐CON‐10

2,4,6‐tris(5‐bromothiophene‐2‐yl)‐1,3,5‐triazine(0.05 g, 8.863 × 10^−5^ mol; Luminescence Technology), (trimethylstannyl thiophen‐2‐yl)benzo[c]^[^ ^1^, ^2^, ^5^ ^]^‐thiadiazole (0.083 g, 1.33 × 10^−4^ mol; Luminescence Technology), tetrakis(triphenylphosphine)palladium(0) (0.0041 g, 4 mol%; Sigma‐Aldrich), and mesitylene (3 mL; Sigma‐Aldrich), all stored in a glove box charged with N_2_ gas, was refluxed at 170 °C for 72 h upon stirring and poured into methanol, and the produced black precipitate was collected by filtration, purified by sequential Soxhlet extraction with methanol, ethanol, dichloromethane, tetrahydrofuran, methanol, ethanol, and acetone (4 h per extraction step), and finally dried in a vacuum for 12 h.

### Na Half‐Cells Assembly and Electrochemical Characterization

The active electrodes were cast on Cu foil by preparing a slurry composed of 70 wt% active material, 20 wt% conductive carbon black, and 10 wt% polyacrylic acid (PAA) in *N*‐methyl‐2‐pyrrolidone (NMP, 99%). This slurry was uniformly applied to the Cu current collector and then dried in a vacuum oven at 100 °C for 12 h. The prepared electrodes were used to evaluate the electrochemical properties of SIB anodes. For electrochemical measurements, CR2032 coin cells were assembled inside a glovebox filled with ultrahigh‐purity argon (99.9999%). The glovebox atmosphere was strictly maintained with H_2_O and O_2_ concentrations below 0.1 ppm. Sodium metal cast on aluminum foil was used as the counter electrode, and a Celgard 3501 separator was employed to separate the anode and counter electrode electrically. The electrolyte solution consisted of 1 m NaPF_6_ dissolved in a propylene carbonate/fluoroethylene carbonate (98:2 w/w) mixture.

Galvanostatic charge–discharge profiles were recorded using a multichannel battery tester (Maccor K4300, Tulsa, OK, USA) within a voltage window of 0.01–2.5 V versus Na/Na^+^. The current density varied from 100 mA g^−1^ to 50 000 mA g^−1^ based on the total weight of the active material. Cyclic voltammograms (CV) were measured at various scan rates (0.1, 0.3, 0.5, 0.8 mV s^−1^) using a multichannel potentiostat (WonATech WMPG1000, Korea) within the same voltage window. Experimental electrochemical impedance spectroscopy (EIS) data were obtained using a single‐channel potentiostat (Oneartec ZIVE SP2, Korea) over a frequency range of 10^−2^ to 10^−5^ Hz at the open‐circuit potential. All electrochemical measurements were conducted in a laboratory environment maintained at 24 °C. For DRT analysis, the MATLAB platform “DRTtools,” developed by Francesco Ciucci and colleagues, was utilized. The code for this analysis can be downloaded from https://github.com/ciuccislab/.^[^ ^36^ ^]^

## Conflict of Interest

The authors declare no conflict of interest.

## Supporting information



Supporting Information

## Data Availability

The data that support the findings of this study are available from the corresponding author upon reasonable request.
