# Supplementary material for: Covalent Organic Nanosheets with a Tunable Electronic Structure to Achieve Unprecedented Stability and High‐Performance in Sodium‐Ion Batteries
Source: Small. 2025 Apr 30;21(36):2502368. doi: 10.1002/smll.202502368 (PMC12423913; doi:10.1002/smll.202502368)
Supplement: Supplementary file 1 — Supporting Information [file SMLL-21-2502368-s001.docx]

Supporting Information

Covalent Organic Nanosheets with a Tunable Electronic Structure to Achieve Unprecedented Stability and High-Performance in Sodium-Ion Batteries

Minseop Lee, Nakyeong Lee, Gumin Kwon, Jae-Min Oh*, Jin Kuen Park*, Seung-Min Paek*

**Contents**

1. Detailed Analysis

2. Figures S1−S11

3. Tables S1−S5

4. Supplementary References

Detailed Analysis

**Peukert constant (**$\boldsymbol{k}$**).** The Peukert effect describes the phenomenon where reaction polarization increases with an increase in the charge/discharge rates, leading to decreased electrochemical performance in energy storage devices.^[2]^ The Peukert constant ($k$) is derived from an empirical law that explains the change in electrochemical performance ($C$) in terms of the charging current density ($I$) and it is expressed using **equation 1**:

$C=C_{0}\times\left( \frac{I}{I_{0}} \right)^{1-k}$ (1)

Where $C$ is the discharge capacity at current density $I$, $C_{0}$ is the discharge capacity at the reference current density $I_{0}$, and $k$ is the Peukert constant. A $k$-value close to the ideal value of 1 indicates a minimal decrease in electrochemical performance with an increasing charge rate. In contrast, higher $k$-values signify increased reaction polarization and more significant capacity loss at high rates.

**CV analysis.** The $b$-value is determined from the slope of the $\logⅈ$ versus $\log v$ graph, which indicates the type of Na^+^-ion storage process. According to **Equations 2** and **3**, the relationship between the peak current ($i$) and the scan rate ($v$) measured at various scan rates can be analysed to calculate the $b$-value.^[3]^

$i=av^{b}$ (2)

$\logⅈ=b\log v +\log a$ (3)

when the $b$-value was 0.5, the electrode follows a fully diffusion-controlled Faradaic mechanism. In contrast, as the $b$-value approaches 1, the electrode exhibits diffusion-independent pseudocapacitive behavior.

The contribution of pseudocapacitance was quantified using the method proposed by Dunn et al. (**Equations 4** and **5**):^[3]^

$ⅈ\left( V \right)=k_{1}v+k_{2}v^{\frac{1}{2}}$ (4)

$\frac{ⅈ\left( V \right)}{v^{\frac{1}{2}}}=k_{1}v^{\frac{1}{2}}+k_{2}$ (5)

Here, the values of $k_{1}$ and $k_{2}$ are determined from the slope and y-intercept of the plot of $v^{\frac{1}{2}}$ versus $\frac{ⅈ\left( V \right)}{v^{\frac{1}{2}}}$. Here, $ⅈ\left( V \right)$ is the current response and $v$ is the scan rate. The term $k_{1}v$ corresponds to the diffusion-independent surface-controlled processes, such as pseudocapacitive or capacitive behaviours, whereas $k_{2}v^{\frac{1}{2}}$ corresponds to the diffusion-controlled processes. This method enables the quantitative distinction of the amount of charge stored at specific potentials from the CV curves.

**Randles-Sevcik equation.** At each scan rate, the cathodic and anodic peak currents ($I_{p}$) displayed a linear relationship with the square root of the scan rate, which can be interpreted using the Randles-Sevcik equation:

$I_{p}=\left( 2.69\times{10}^{5} \right)\cdot n^{1.5}\cdot S\cdot D_{{Na}^{+}}^{0.5}\cdot v^{0.5}\cdot C_{{Na}^{+}}$ (6)

where $I_{p}$ is the peak current ($A$), $n$ is the number of electrons transferred, $S$ is the electrode area (1.13 cm^2^), $C_{{Na}^{+}}$ is the concentration of Na^+^-ions in the electrolyte (mol cm^−3^), $v$ is the scan rate (V s^−1^), and $D$ is the diffusion coefficient (cm^2^ s^−1^).^[4]^

**EIS analysis.** The Nyquist plot derived from the experimental EIS data reflects a combination of multiple resistance processes, making it challenging to isolate individual internal resistances across various charge states and to track the evolution of resistance components during cycling. To address this complexity, we conducted a time-scale analysis of the impedance data to better understand the dynamic behavior and trends of internal resistance components after cycling. For the decomposition and analysis of impedance data under different cycling conditions, we utilized the MATLAB-based 'DRTtools' package, developed by Francesco Ciucci and colleagues.^[5]^ In the DRT representation, each feature is characterized by a time constant associated with the $RC$ element in time ($\tau_{n} = R_{n}C_{n} = \frac{1}{2}\pi f_{n}$). Additionally, the equivalent circuit model (**Figure 6i**) fitting parameters derived from the time-scale-based impedance data are summarized and compared in **Tables S4** and **S5**.

In the equivalent circuit model, each resistance component was classified as Rs (ohmic resistance), $R_{c}$ (film resistance), $R_{ct}$ (charge transfer resistance), and $R_{ion}$ (ionic resistance). The $R_{s}$ primarily represents the contact resistance of the electrode arising from physical contact between the electrode and the current collector; $R_{f}$ is related to the SEI layer formed owing to electrolyte reactions on the electrode surface; $R_{ct}$ represents the charge transfer resistance at the interface, associated with electron movement between the electrode and the electrolyte; and $R_{ion}$ mainly denotes the resistance originating from the structural deformations of the electrode material and the electrolyte trapped in the pores of the composite electrode during continuous cycling.

**
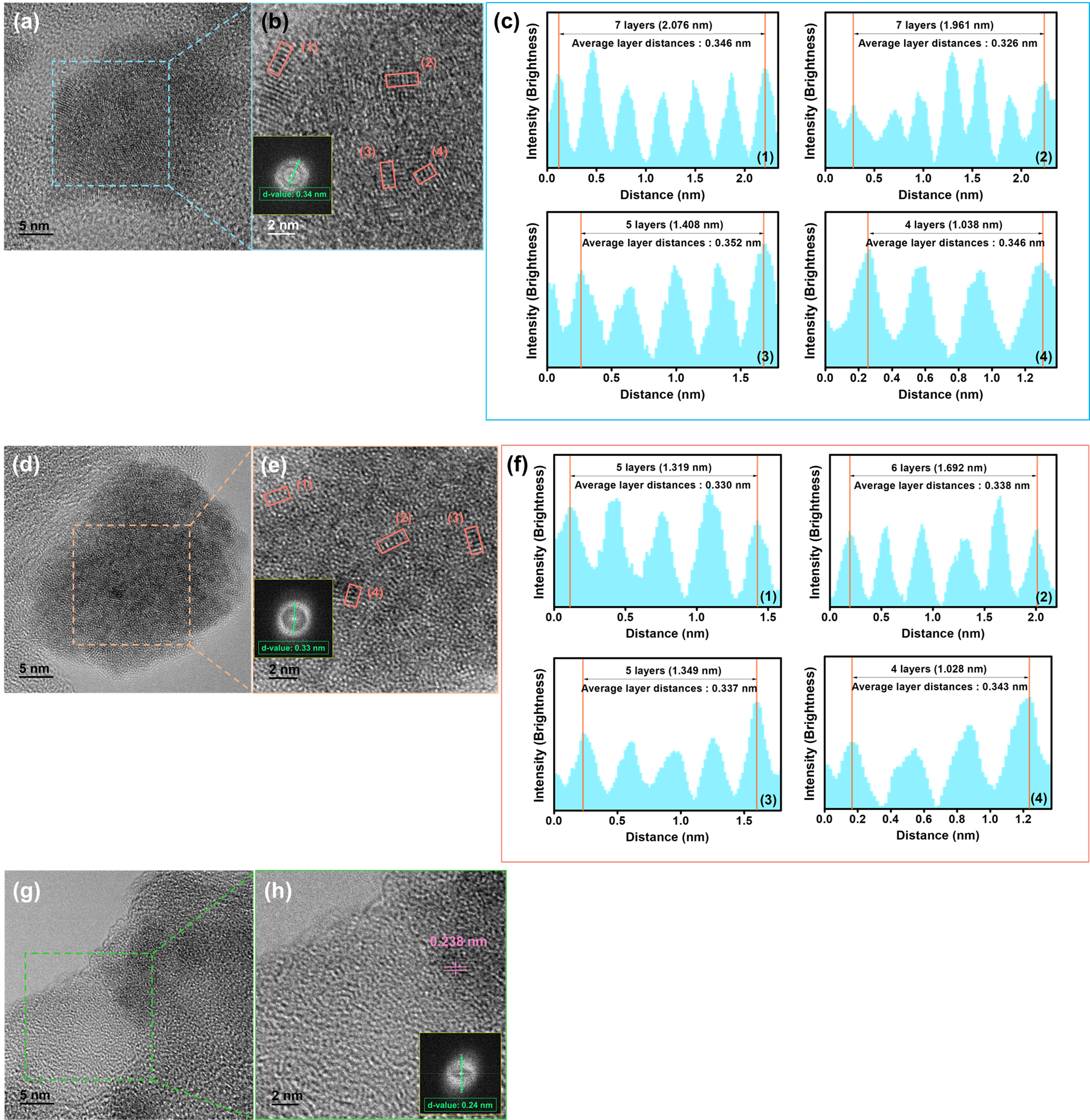
**

**Figure S1**. (a) High resolution (HR) TEM image of the crystalline domain of D/A-CON-10-F; (b) The fast Fourier transform (FFT) pattern (the inset image) at the selected area with a blue-coloured and dotted rectangle in (a) and its inverse FFT images; (c) Brightness profiles of the selected areas; (d) HRTEM image of the crystalline domain of D/A-CON-10-F; (e) The FFT pattern (the inset image) at the selected area with yellow-coloured and dotted rectangle in (d) and its inverse FFT images; (f) Brightness profiles of the selected areas; (g) HRTEM image of the crystalline domain of D/A-CON-10-F; (h) The FFT pattern (the inset image) at the selected area with green-coloured and dotted rectangle.

Some crystallites with a d-spacing of approximately 0.24 nm were also observed (**Figure S1h**), which are consistent with the (200) plane of SnO_2_, indicating that trace amount of Sn remained as a residue even after the purification process. However, the size of trace SnO_2_ would be too small to be detected by PXRD (**Figure 1h**) or they were amorphous.


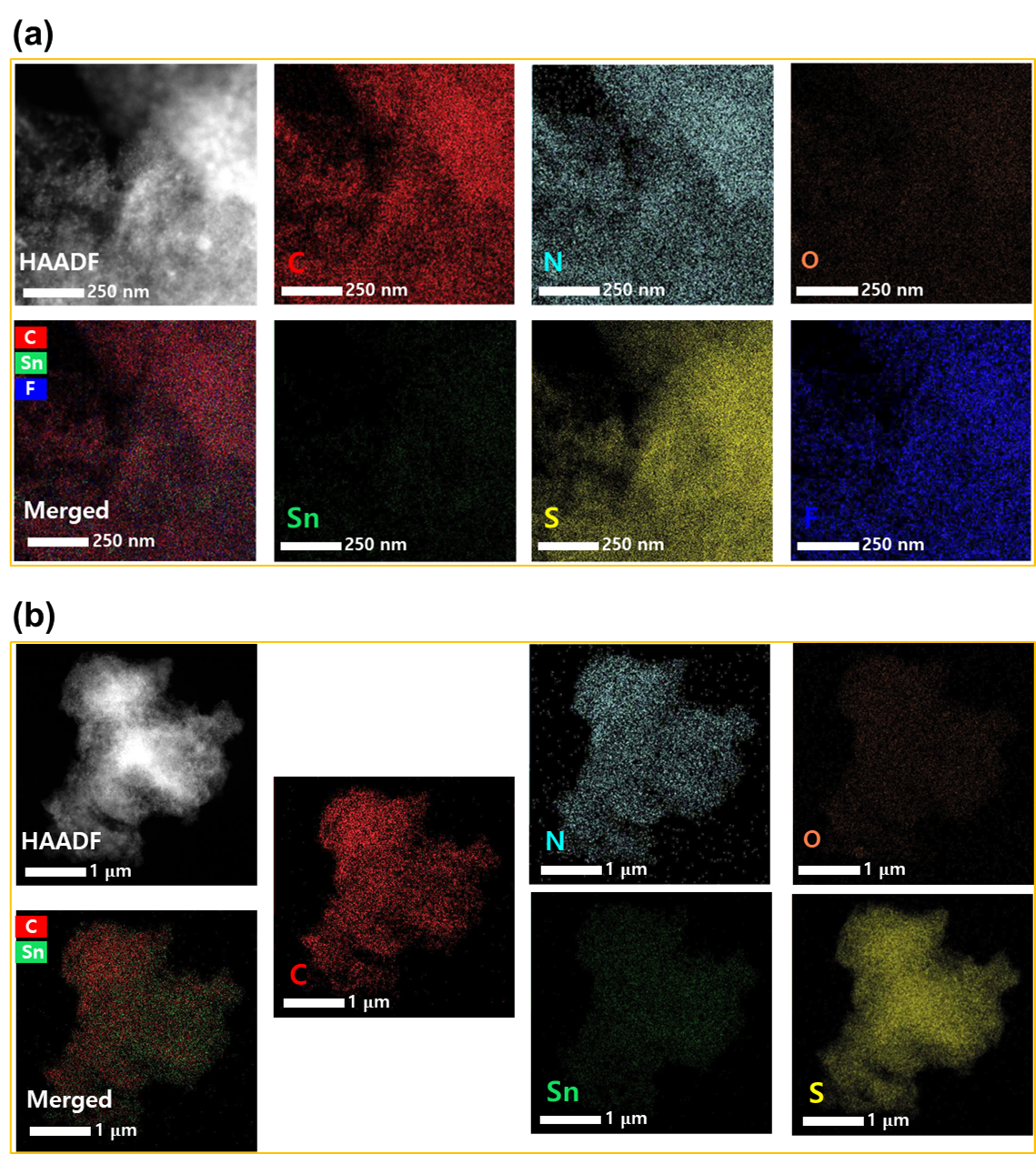
**Figure S2.** High-Angle Annular Dark-Field Scanning Transmission Electron Microscopy (HAADF STEM) images and energy dispersive X-ray spectroscopy (EDS) elemental distribution maps of (a) D/A-CON-10-F and (b) D/A-CON-10.

**
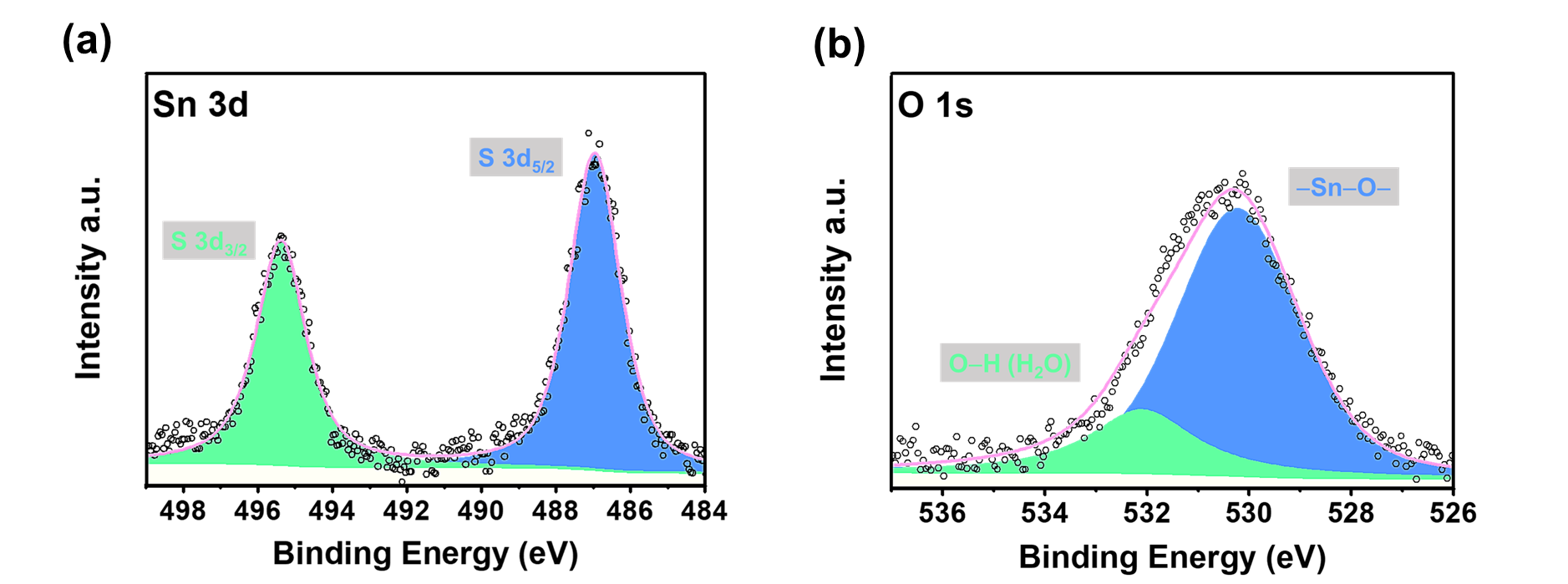
Figure S3.** High-resolution XPS profiles of (a) Sn 3d, and (b) O 1s of D/A-CON-10-F.

**
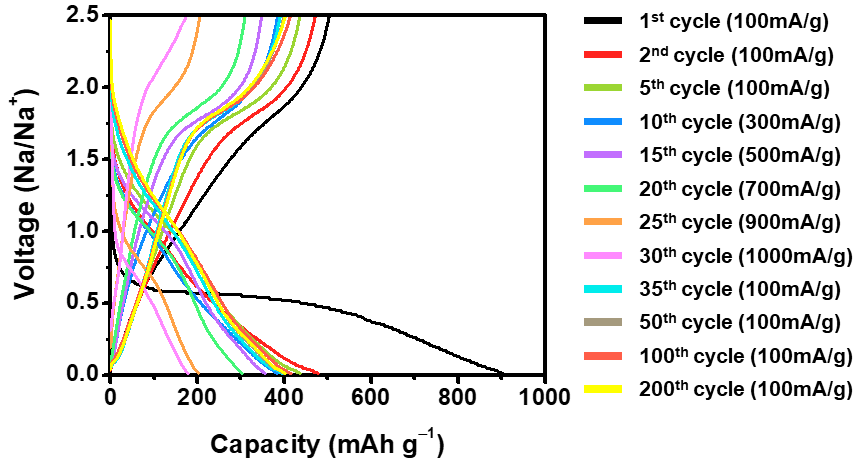
 Figure S4.** Galvanostatic charge-discharge (GCD) profiles of D/A-CON-10 electrode at different current densities (100–1000 mA g^−1^).

**
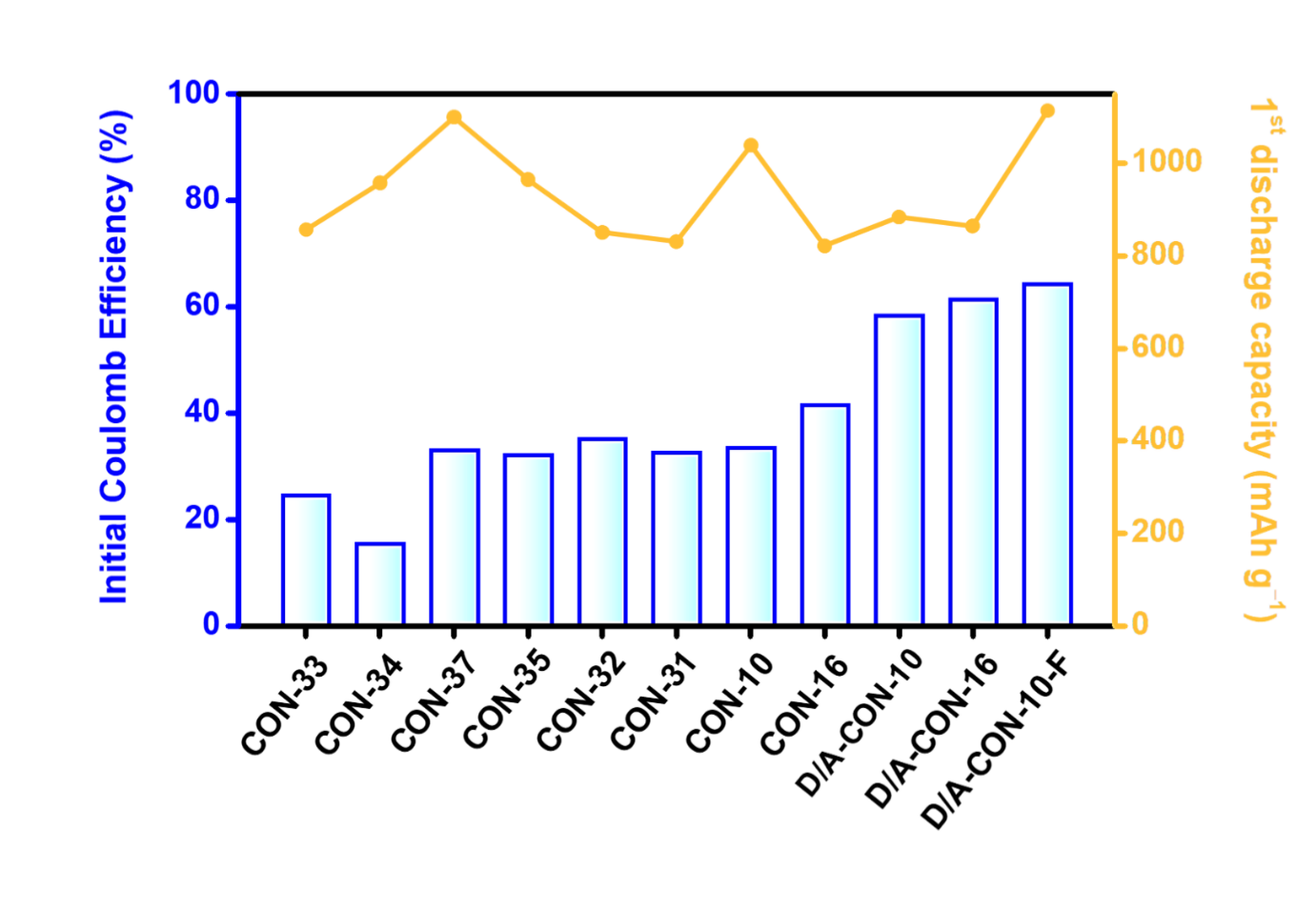
Figure S5.** 1^st^ discharge capacity and initial Coulombic efficiency (ICE) comparison of D/A-CON-10-F electrode vs. other CON-based electrodes.^[1,6,7]^


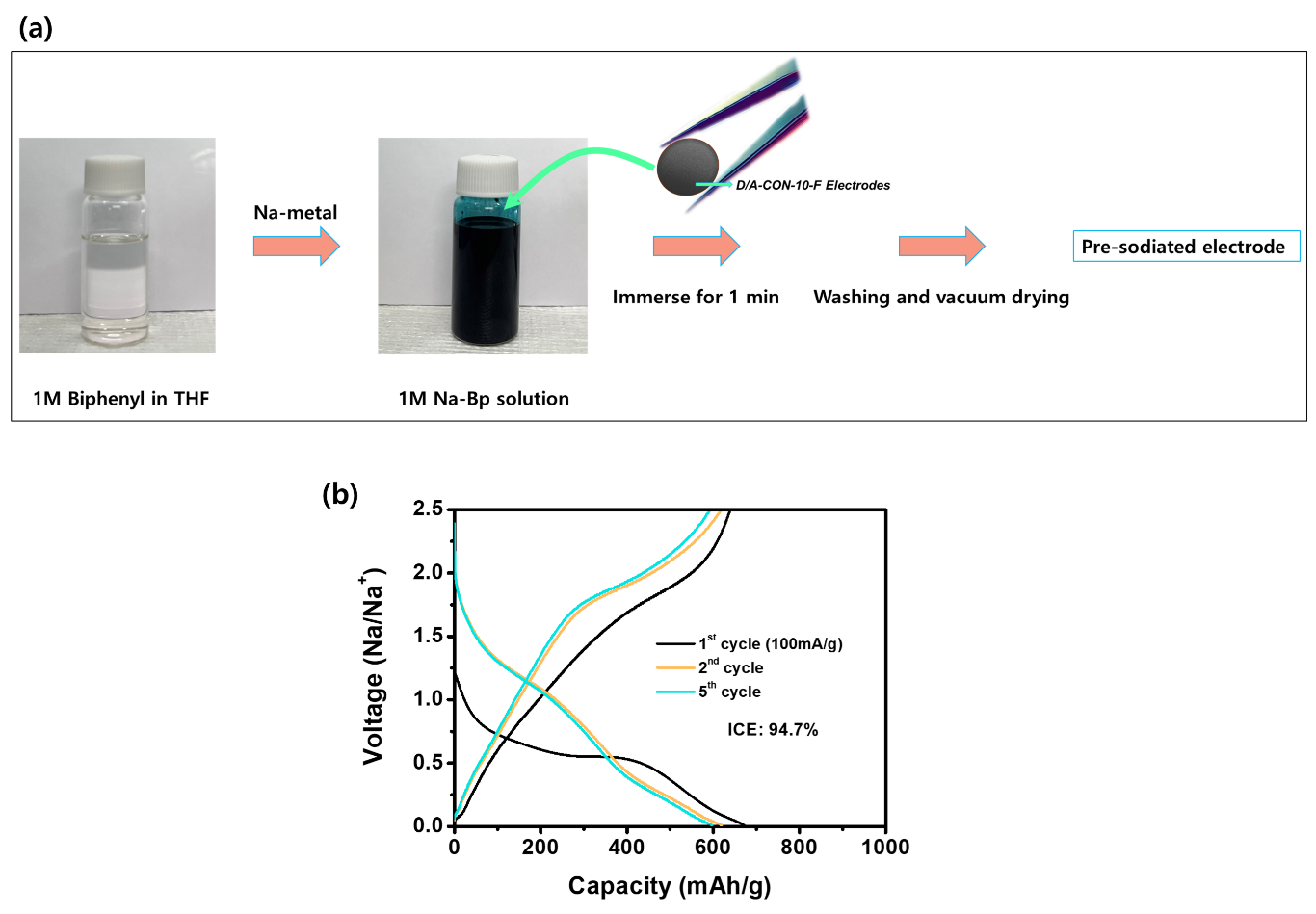


**Figure S6.** (a) Chemical pre-sodiation process of the D/A-CON-10-F electrode; (b) GCD profile and ICE of the chemically pre-sodiated D/A-CON-10-F electrode.

**Figure S6a** illustrates the pre-sodiation process performed on the D/A-CON-10-F electrode. First, a 1 M solution of Na-biphenyl (Na-Bp) is prepared by dissolving biphenyl in THF (Tetrahydrofuran) at a concentration of 1 M and reacting it with Na-metal inside an argon-filled glove box. This process is conducted under strictly anhydrous and oxygen-free conditions to prevent side reactions of sodium metal with moisture or oxygen in the solution. Subsequently, the D/A-CON-10-F electrode is immersed in the prepared Na-Bp solution for approximately 1 minute to induce chemical pre-sodiation. During this process, Na^+^-ions infiltrate the surface and interior of the electrode, pre-completing a portion of the irreversible reactions that would typically occur during the initial cycling. After immersion, the electrode is rinsed with THF to remove residual sodium salts or biphenyl byproducts from the surface. The cleaned electrode is then subjected to vacuum drying for approximately 12 hours to ensure complete removal of any remaining solvent. The pre-sodiated electrode is assembled into a Na half-cell to evaluate its ICE and capacity performance. The results confirm that the pre-sodiation process significantly reduces irreversible capacity loss and substantially improves the ICE of the D/A-CON-10-F electrode.

**
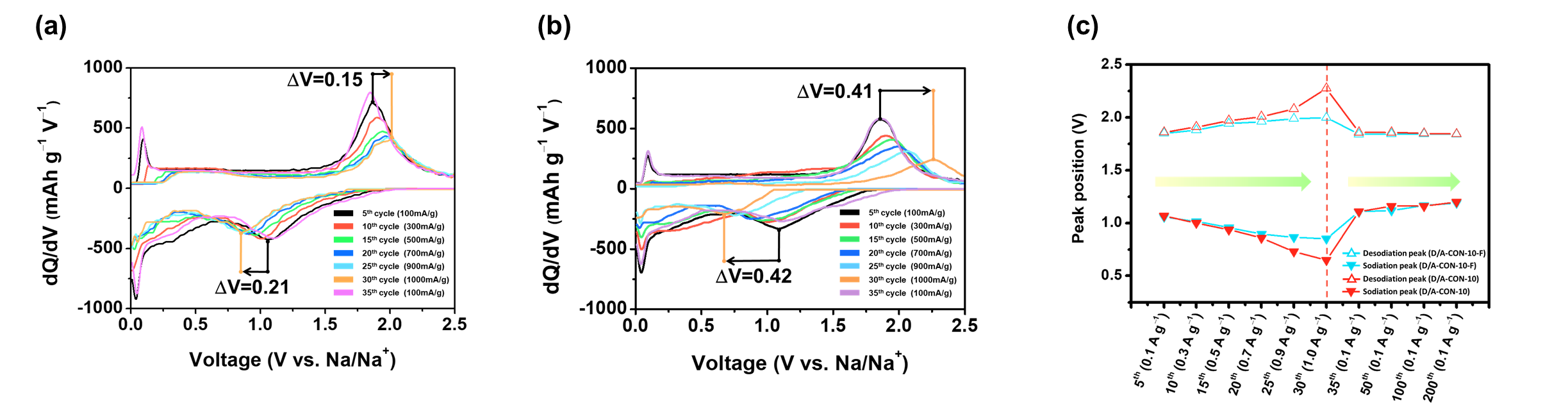
Figure S7.** dQ/dV curves of (a) D/A-CON-10-F and (b) D/A-CON-10 derived from the charge-discharge profiles. (c) Comparison of polarization as current density varies.

**
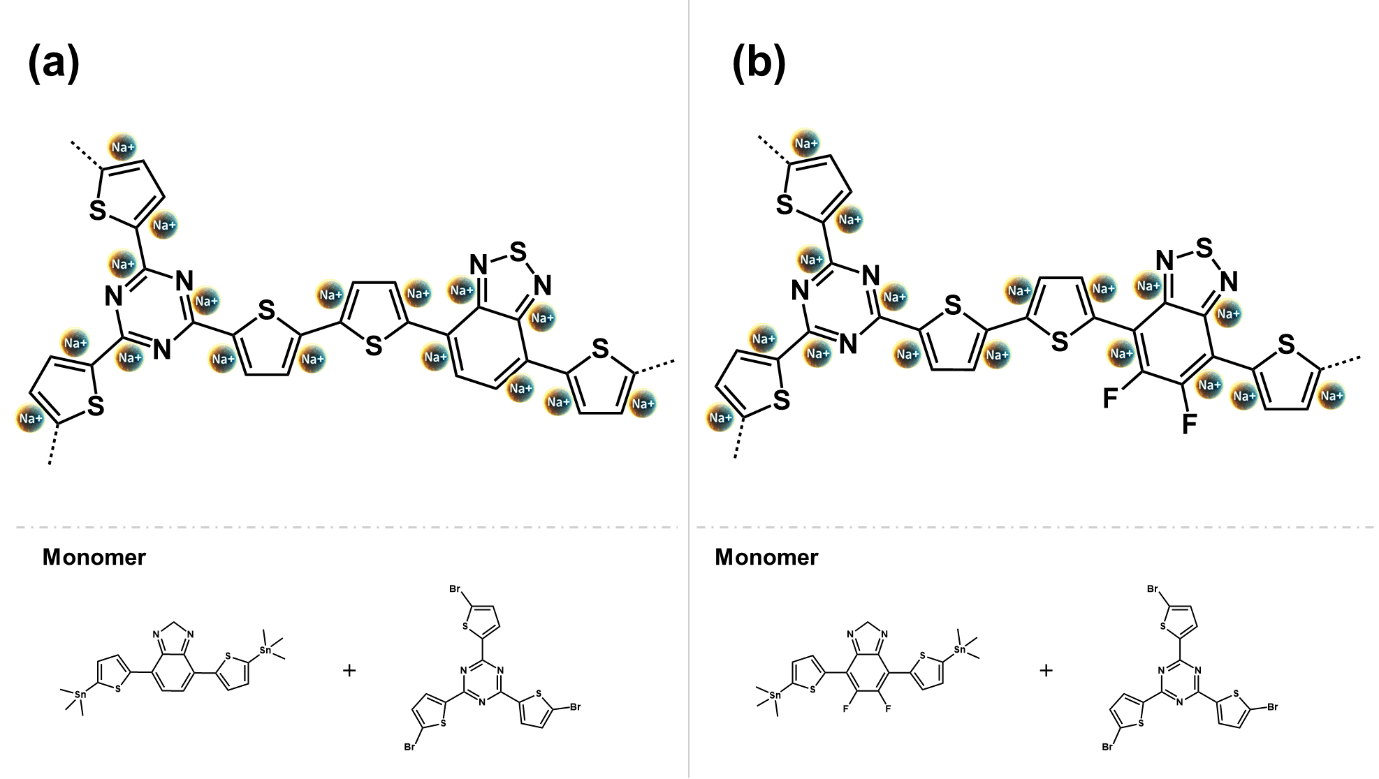
Figure S8.** The schematic illustration of Na-ion binding models for CONs. The structures represent unit structures of a) D/A-CON-10 and b) D/A-CON-10-F and blue-colored balls denote Na+-ions.

The repeating units of D/A-CON-10-F and D/A-CON-10 contain 17 double bonds (C=C, and C=N), which act as redox-active sites for Na^+^-ion storage. The chemical formula of the repeating unit for D/A-CON-10-F is C_28_H_10_N_5_F_2_S_6_, with a calculated molecular weight of 646.85 g mol^–1^. One mole of Na-ions provides one mole of electrons, and one mole of electrons corresponds to a charge of 96485 C. Therefore, the total charge provided by 17 Na^+^-ions is calculated as follows:

17×96485 C mol^–1^=1640245 C mol^–1^

The theoretical capacity (C) is calculated as follows:

C=1640245 C mol^–1^ / 646.85 g mol^–1^ ≈ 2535.26 C g^–1^

To convert this to mA h/g, we divide by the number of seconds in an hour (3600 s):

Capacity=2685.87 C g^–1^ / 3600 s h^–1^ ≈ 704.24 mA h g^–1^

Therefore, the theoretical capacity of D/A-CON-10-F is approximately 704.24 mA h g^–1^. The theoretical capacity of D/A-CON-10, calculated using the same process, is approximately 745.52 mA h g^–1^.

**
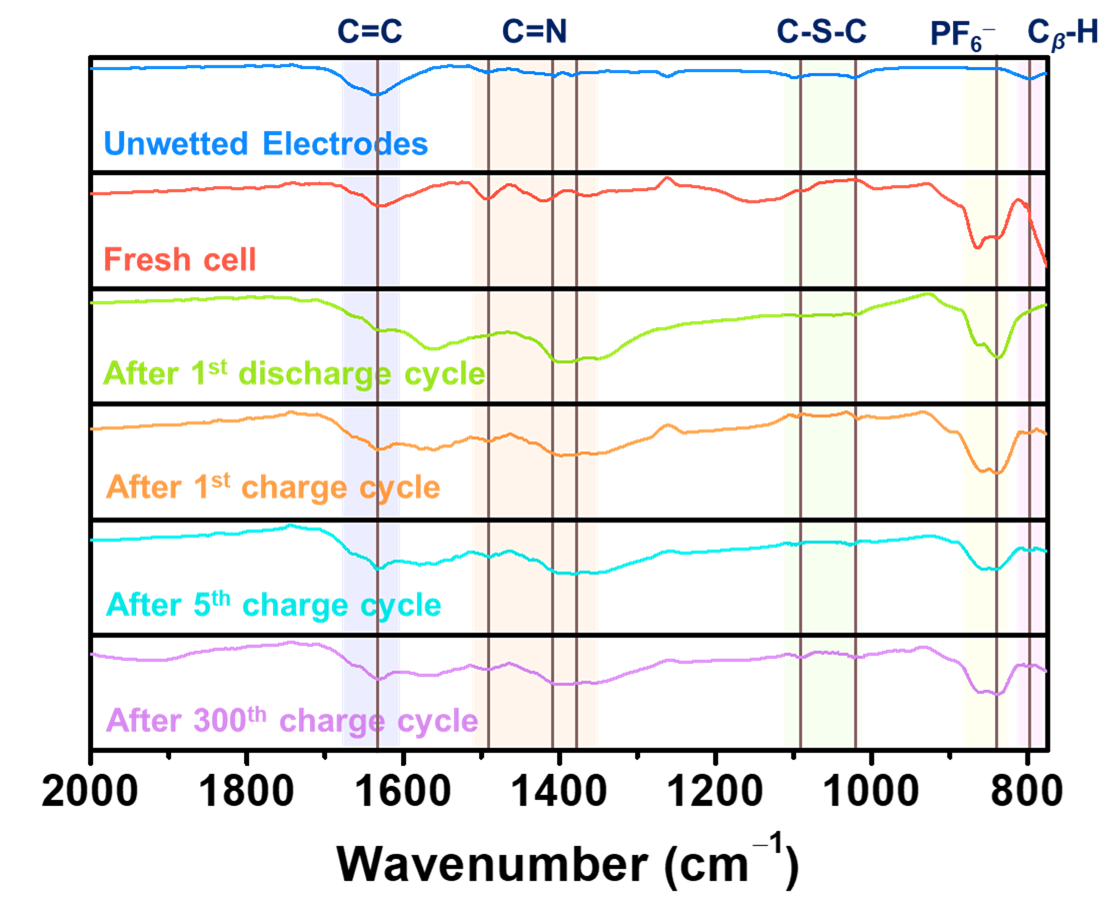
**

**Figure S9.** Ex-situ FTIR spectra of D/A-CON-10-F electrode.

**
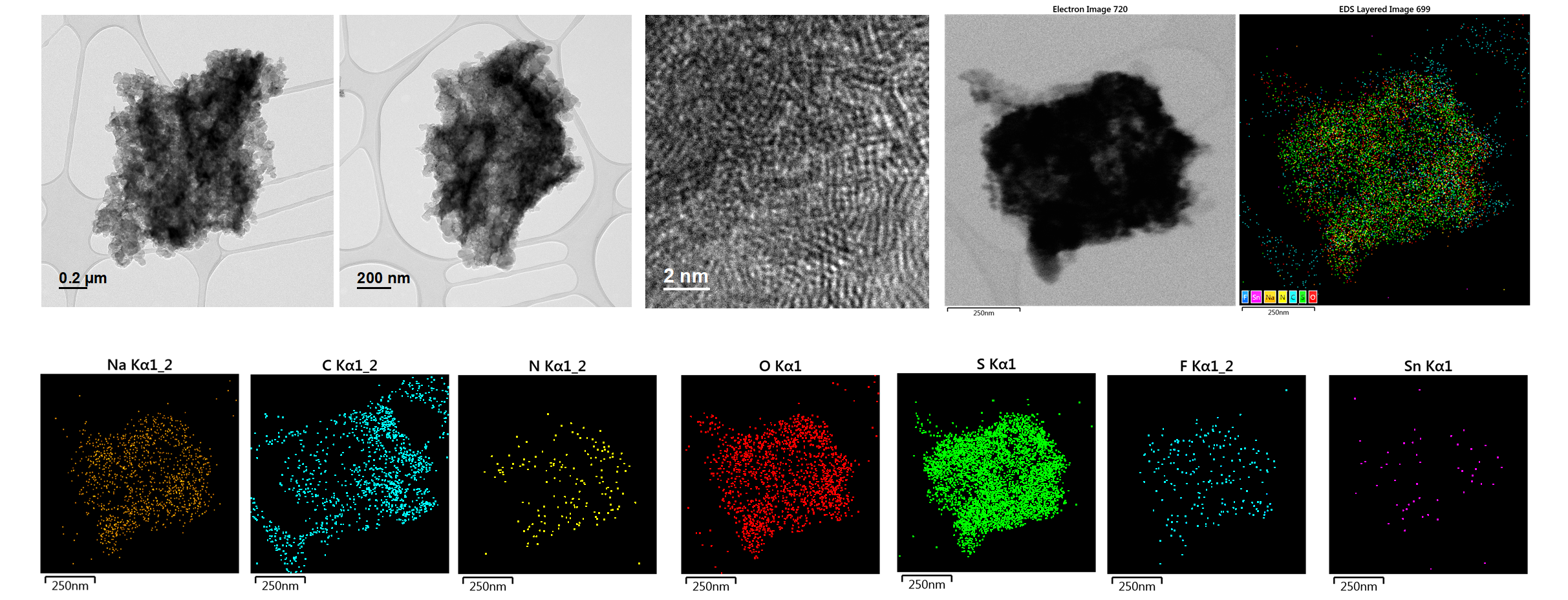
**

**Figure S10.** Ex-situ (HR)TEM and EDS images of D/A-CON-10-F electrode after the 300^th^ charge cycle.

**
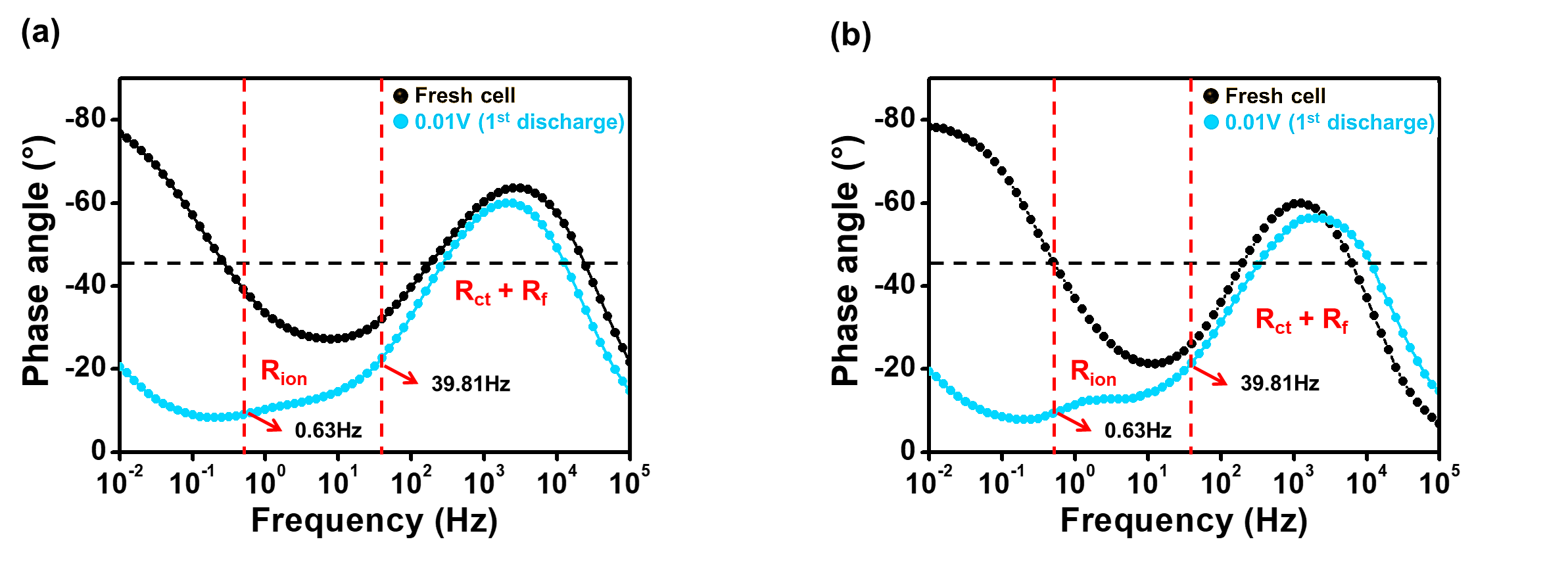
**

**Figure S11.** Bode plots of the (a) D/A-CON-10-F and (b) D/A-CON-10 electrodes measured during the initial cycle.

**Table S1.** Elemental analysis of D/A-CON-10-F.

| **XPS (at%)** | | | | | | **XPS (wt%)** | | | | | |
| --- | --- | --- | --- | --- | --- | --- | --- | --- | --- | --- | --- |
| **C** | **N** | **O** | **S** | **F** | **Sn** | **C** | **N** | **O** | **S*** | **F** | **Sn*** |
| 68.39 | 9.33 | 5.06 | 11.63 | 5.1 | 0.49 | 52.62 | 8.37 | 5.19 | 23.89 | 6.21 | 3.73 |

The repeating unit of D/A-CON-10-F is C_28_H_10_N_5_F_2_S_6_, and the mass ratios of sulfur in the repeating unit is 29.74%.

In SnO_2_ (150.71 g mol^–1^), the mass fraction of Sn (118.71 g mol^–1^) is 78.78% (118.71/150.71).

According to the XPS analysis, the mass fractions of S and Sn are 23.89 wt% and 3.73 wt%, respectively. ICP result with 4.05 wt% of Sn is also in good agreement with XPS result.

Thus, considering only S and Sn, the mass ratio is approximately 6.40:1.

The molar ratio of D/A-CON-10-F units (calculated from S wt%) to SnO_2_ (calculated from Sn wt%) can then be determined as follows:

(192.39 × *n*CON)/(118.71 × *n*SnO_2_) = 6.40

nCON/*n*SnO_2_ = 6.40×(118.71/192.39) = 3.95

Therefore, the molar ratio of D/A-CON-10-F units to SnO_2_ is approximately 3.95 mol:1 mol.

The overall mass ratio of D/A-CON-10-F units to SnO_2_ is approximately 94.4:5.6 (wt%).

**Table S2.** Binding energy position, full width at half maximum (FWHM) and area of each core peak of D/A-CON-10-F.

| **Core levels** | **D/A-CON-10-F** | | |
| --- | --- | --- | --- |
|  | **Binding energy/eV** | **FWHM/eV** | **Area** |
| **C 1s** | 284.4 | 1.6 | 17974.6 |
|  | 286.2 | 1.7 | 6946.1 |
|  | 288.0 | 2.2 | 1378.8 |
|  | 289.2 | 1.7 | 1016.4 |
|  | 290.7 | 2.0 | 700.5 |
| **N 1s** | 398.0 | 1.5 | 3729.7 |
|  | 399.1 | 1.1 | 1467.7 |
|  | 399.8 | 1.5 | 1021.9 |
|  | 401.2 | 2.5 | 908.6 |
| **O 1s** | 530.2 | 2.8 | 5256.5 |
|  | 532.1 | 2.5 | 1382.7 |
| **S 2p** | 163.2 | 1.2 | 5283.4 |
|  | 164.4 | 0.8 | 933.3 |
|  | 164.7 | 1.0 | 3330.1 |
|  | 165.7 | 1.6 | 1339.4 |
| **F 1s** | 686.3 | 1.3 | 4738.3 |
|  | 687.3 | 2.9 | 3366.6 |
| **Sn 3d** | 486.9 | 1.6 | 4685.1 |
|  | 495.4 | 1.6 | 3390.3 |

**Table S3.** Comparison of electrochemical sodium storage performance of organic anodes reported since 2020; a (mA h g^–1^); b (mA g^–1^).

| **Anode materials** | **Synthesis Method** | **Novelty & Strategy** | **Performance Metrics (Capacity^a^/Current density^b^/Cycle number)** | **Voltage range (vs. Na/Na^+^)** | **Ref.** |
| --- | --- | --- | --- | --- | --- |
|  |  | **Key Features** |  |  |  |
| **D/A-CON-10-F** | Stille cross-coupling reaction incorporating fluorine into BT-based framework | -Combined BT motifs and fluorine for reduced bandgap and electron density | **637/100/500**  **375/1000/5000**  **207/5000/5000**  **97/10000/5000**  **75/20000/5000** | **0.01–2.5** | **This work** |
|  |  | -High Na^+^ ions storage, improved conductivity, and stable cycling up to 5000 cycles |  |  |  |
| **D/A-CON** | Solvothermal Stille cross-coupling and reflux methods | -Inclusion of electron-deficient benzothiadiazole (BT) units for bandgap-dependent Na^+^ storage  -Donor/acceptor conjugation to enhance conductivity | D/A-CON-10: ~450/100/30  D/A-CON-16:  ~534/100/30 | 0.01–2.5 | [1] |
|  |  | -High conductivity, large surface area, high Na^+^ insertion sites, tunable porosity |  |  |  |
| **Fluorinated covalent organic nanosheet (CON-37)** | Cross-coupling using Pd(0) catalyst (Stille coupling reaction). | -Introducing fluorine atoms to modify dipoles  -Increasing the polarity of the CON framework | 350.7/100/2500 | 0.01–2.5 | [6] |
|  |  | -Improved structural stability, electrical conductivity  -High capacity, and superior ionic/electrical conductivity. |  |  |  |
| **CON-G-10** | Solvothermal synthesis using Stille cross-coupling reaction with CONs and graphene nanosheets (GNs) | -Hybridization of CONs with GNs via solvothermal method  -Enhanced electrical conductivity through well-overlapped hybrid structure | 295/100/30 | 0.01–2.5 | [8] |
|  |  | -High electrical conductivity, improved Na^+^ accessibility, well-overlapped structure |  |  |  |
| **p-TSA-PPy** | Chemical oxidation with p-toluenesulfonic acid doping | -Conductive polymer with high Na^+^ storage  -Doping with p-toluenesulfonic acid to improve conductivity | 185/760/250  135/3800/250  120.5/760/2000 | 0.1–3.2 | [9] |
|  |  | -High conductivity  -Stable sponge-like structure |  |  |  |
| **DAAQ–HCCP COF** | Condensation via sonication | -Incorporation of redox-active quinones with a hexachlorocyclotriphosphazene (HCCP) linker containing six replaceable sites  -Replace all -Cl sites of HCCP with redox-active DAAQ to form a stable framework | 209/100/1  88/100/100  72/2000/1000 | 0.01–2.0 | [10] |
|  |  | -Stable framework  -Ordered porous structure  -Increased redox-active sites due to complete replacement of -Cl sites  -Nanosheet structure with uniform size distribution |  |  |  |
| **IISERP-COF18** | Polycondensation reactions and DFT optimization | -Varying LUMO energy levels by including electron-deficient centers in COF structure  -Manipulating LUMO levels through electron-deficient centers (phenyl vs. tetrazine vs. bispyridine-tetrazine) | 410/100/-  340/1000/1400  178/10000/-  128/15000/- | 0.05–3.0 | [11] |
|  |  | -Enhanced specific capacity and rate performance for Na^+^ ions through electronic driving force and intrinsic porosity |  |  |  |
| **Sodium 5,5′-carbonylbis(isobenzofuran-1,3-dione) (SCID)** | Recrystallization and vacuum solution impregnation with MWCNTs | -Bridging carbonyl group activating ortho-carbonyl groups  -Incorporation of bridging carbonyl group and multi-walled carbon nanotubes (MWCNTs) | SCID:  158/50/100  SCID@CNT: 182/50/100 | 0.01–3.0 | [12] |
|  |  | -High theoretical specific capacity, enhanced Na^+^ insertion, improved conductivity with MWCNTs |  |  |  |

| **Trisodium 1,2,4-benzenetricarboxylate (TBC)** | Neutralization with NaOH in ethanol | -Introducing three carboxylate groups to stabilize the anode and reduce solubility in electrolyte | 195/50/1  137/50/600 | - | [13] |
| --- | --- | --- | --- | --- | --- |
|  |  | -Low cost, high sustainability, high capacity, long cycle life |  |  |  |
| **Sodium thieno[3,2-b]thiophene-2,5-dicarboxylate (STTDC)** | Bromination, cyanation, and acidification | -Thiophene backbone to enhance charge transfer and electron conductivity | 430/50/40  288/2000/4000 194/5000/4000 | 0.01–3.0 | [14] |
|  |  | -High electron transfer capability, excellent cycling stability, high rate capability |  |  |  |
| **PQL (Polyazaacene)** | Reaction between carbonyl, hydroxyl groups of 2,5-dihydroxy-1,4-benzoquinone and amino groups of tetraaminobenzene tetrahydrobromide in polyphosphoric acid | -Stable large conjugated ladder structure  -Polymerization of organic small molecules into polymers with stable frameworks | 317/100/400 | 0.01–3.0 | [15] |
|  |  | -High theoretical specific capacity  -Strong structural designability  -Relatively inexpensive manufacturing cost |  |  |  |
| **DAB-NiPc** | Catalyst-free coupling reaction between 4-nitronickel phthalocyanine and 4,4′-diaminobiphenyl | - Largest pore size with highest surface area | 395/200/1000 | 0.01–3.0 | [16] |
|  |  | -Pore size: 2.74 nm  -Surface area: 575 m²/g  -Excellent cycling stability and rate capability |  |  |  |
| **Sodium 5-(4-carboxyphenyl)picolinate (Na-CPP)** | Alkalization reaction between 5-(4-carboxyphenyl)picolinic acid and NaOH | -Introduction of N atoms in phenylpyridine moiety to improve electronic properties and reduce polarization | 197/100/350 | 0.01–2.5 | [17] |
|  |  | -Smaller particle size for better ion transport  -Stable redox behavior, high capacity retention |  |  |  |
| **Polymeric Carbon Nitride (PCN)** | Polycondensation followed by moderate pyrolysis at 350 °C | -Moderate pyrolysis to reduce optical bandgap and tune microstructures | 351/30/-  296/150/- | 0.01–3.0 | [18] |
|  |  | -High specific capacity  -Superb rate capability, and ultrastable cyclability with reduced conductive carbon additive |  |  |  |
| **2,2′-Bipyridine-5,5′-dicarboxylic acid (H_2_bpy)** | Direct use of commercially available H_2_bpy | -Introducing conjugated carboxylic acid-based heterocyclic structure | 258/200/100 | 0.01–3.0 | [19] |
|  |  | -High specific capacity, and remarkable rate performance in both lithium- and sodium-ion batteries |  |  |  |
| **CuPc-2D-cCOFs** | Solvent-free synthesis using metal salts (e.g., Cu(OAc)_2_·H_2_O) and 1,2,4,5-benzenetetranitrile (BTN) at 350°C | -2D conjugated structure with π-stacking layered structure and N-rich pore channels | 538/50/-  342/1000/-  325/1000/2500 | 0.01–3.0 | [20] |
|  |  | -High Na-ion storage capacity with N-rich phthalocyanine units and conjugated pyrrole moieties  -High reversible capacities, good rate performance, excellent stability |  |  |  |
| **Sodium 1,10-phenanthroline-3,8-dicarboxylate (S-PD)** | Neutralization of 1,10-phenanthroline-3,8-dicarboxylic acid (H2-PD) with sodium hydroxide in ethanol | -Large π-conjugated planar structure  -Introduction of heteroatoms (N) for enhanced conductivity and additional Na^+^ storage | 210/50/100  103/515.4/500 | 0.01–2.6 | [21] |
|  |  | -High discharge capacity, excellent diffusion ability  -Fast reaction kinetics, stable S-PD/electrolyte interface |  |  |  |

| **Covalent Organic–Inorganic Hybrid Polymer (COIHP-1)** | Condensation reaction of tris(2,3,6,7,10,11-hexahydroxytriphenylene) and hexachlorophosphazene in a 1:1 mesitylene-dioxane solution | -Uses a hybrid organic-inorganic framework combining redox-active 2D organic materials with inorganic phosphazene. | 310/35/-  199/35/500 | 0.01–3.0 | [22] |
| --- | --- | --- | --- | --- | --- |
|  |  | -High electrical conductivity (9.52 × 10⁻³ S/cm)  -Mesoporous structure, reversible Na+ storage, stable cycling performance |  |  |  |
| **PN (mixture of CPP and CPN isomers)** | Mixed in different proportions, alkalized. CPP: CPN = 3:1 (mass/molar ratio) showed optimal performance. | -First example of mixing two isomers as organic anode materials in SIBs.  -Mixing isomers to form uniform spherical particles | 204.6/100/-  154/935/1200 | 0.01–3.0 | [23] |
|  |  | -Higher Initial Coulombic Efficiency (ICE) of 85%  -Ultra-stable reversible capacity, 92% capacity retention after 1200 cycles at 935 mA/g. |  |  |  |
| **Disodium 2,5-difluoroterephthalate (DFTP-Na)** | Halogenated carboxylate synthesis using 2,5-difluoroterephthalic acid dispersed in ethanol with sodium hydroxide. | -Fluorinated carboxylate anode outperforms other halogenated carboxylates (Cl, Br) and non-halogenated carboxylates. | 161/50/300  103.2/1000/1000  236/20/-  81/5000/- | 0.01–2.0 | [24] |
|  |  | -Introducing F atoms to reduce solubility, enhance cyclic stability, and interact with Na^+^ during redox reactions. |  |  |  |
| **2,9-Dimethylquinacridone (2,9-DMQA)** | Quinacridone (QA) and 2,9-dimethylquinacridone (2,9-DMQA) were pyrolyzed at 600 °C under an N_2_ atmosphere. | -High char yield (61% at 600°C), morphological development influenced by crystal orientation  -Pyrolysis of quinacridone derivatives, exploiting parallel-oriented crystal structures for morphological control. | 247/100/200  134/1000/5000 | 0.01–3.0 | [25] |
|  |  | -High char yield  -Growth of longitudinal polycyclic aromatic hydrocarbons (PAHs) and formation of disordered structures due to gas evolution. |  |  |  |
| **Azatriangulenetrione** | Synthesized through Ullman coupling, hydrolysis, and ring closure reactions. | -Utilizing lone pair electrons of nitrogen for intramolecular radical stabilization. | 308/100/250 | 0.01–3.0 | [26] |
|  |  | -Reversible four-step one-electron redox chemistry  -Stabilized radical species  -High cycling stability |  |  |  |
| **Na_2_dmcdbq-CNT composite** | In situ growth of carbomethoxy-modified quinone-based disodium salt on CNTs | -Introduction of carbomethoxy groups for enhanced ionic diffusion  -In situ growth on CNTs | 151/4000/6000  86/7160/35000 | 0.01–2.5 | [27] |
|  |  | -High ionic diffusion  -Excellent rate performance, long cycle stability |  |  |  |
| **4,4′-Biphenyldicarboxylic acid (H_2_bpdc)** | Commercially available, used without purification | -Conjugated aromatic acid as anode for SIBs  -Conversion-type mechanism | 215/100/300 | 0.01–2.5 | [28] |
|  |  | -High theoretical specific capacity  -Explored in esters and ethers-based electrolytes  -Layered crystal structure |  |  |  |
| **Polymeric disodium phthalocyanines (pNaPc)** | One-pot cyclization and polymerization of bisphthalonitriles (O- and S-linked) with NaOC_5_H_11_ in 1-pentanol | -Novel polymeric anodes with tunable linkers for SIBs  -Linker type variation (O- and S-linkers) | O-pNaPc:  96.0/200/200  S-pNaPc:  94.6/200/200 | 0.01–2.0 | [29] |
|  |  | -High capacity, excellent cycle stability  -Enhanced redox kinetics  -Structural stability, high energy density |  |  |  |
| **BCOF-1 (Benzimidazole-linked COF)** | Condensation reaction between hexaazatrinaphthalenehexamine (HATNHA) and terephthalaldehyde (TA) | -Hexaazatrinaphthalene cores linked by imidazole moieties | 365/40/-  311/400/-  249/1200/-  196/2000/-  136/3200/-  104/4000/-  50/6000/- | 0.01–3.0 | [30] |
|  |  | -High surface area  -High theoretical specific capacity  -Mesoporous channels  -Pseudocapacitive behavior |  |  |  |

| **PPD (Porous Polyimide)** | Polycondensation of pyromellitic dianhydride (PMDA) and 2,6-diaminoanthraquinone (DAAQ) | -Introduction of multicarbonyl active centers  -Porous structure | 244.6/100/100  100.7/1000/2000 | 0.01–3.0 | [31] |
| --- | --- | --- | --- | --- | --- |
|  |  | -High conductivity  -Environmentally friendly  -High thermal stability  -Large contact area with Na-ions |  |  |  |
| **Sulfur-Containing Carbonaceous Anode Material (CS)** | Bottom-up synthesis using thermal treatment | -Precise control over sulfur content  -Doping with sulfur to enhance electrochemical performance | 497/100/1  450/100/100  290/1000/200  703/50/-  508/100/-  416/200  324/500/-  255/1000/- | 0.1–3.0 | [32] |
|  |  | -High sulfur content  -Controlled sulfur distribution  -Amorphous structure with large interplanar distance |  |  |  |
| **DBD-CMP2** | Suzuki polycondensation of DBD-4Bin and tribromobenzene | -Thiophene-rich conjugated microporous polymer for Na storage  -Increasing cross-linked degree and thiophene content | 414/50/-  241/100/100 | 0.005–3.0 | [33] |
|  |  | -Fully conjugated skeleton  -High thiophene content  -Low LUMO energy level  -Narrow band gap |  |  |  |
| **TQA** | Condensation reaction of hexaketocyclohexane and o-phenylenediamine | -Using DFT calculations to identify active sites  -Adding sodiated Nafion to improve stability | 135/350/100 | 0.01–2.5 | [34] |
|  |  | -High-density redox-active pyrazine sites  -Formation of intermediate radical anions  -Enhanced cycling stability with sodiated Nafion layer |  |  |  |
| **Sodium benzene-1,2,4-tricarboxylate (Na-1,2,4-BTC)** | Neutralization reaction | -Asymmetric carboxylic-based coordination salt for Na storage  -Stable asymmetric framework, enolization mechanism, fast charge transfer | 157.3/25/50  163/100/-  153.9/200/-  139.6/500/-  111.1/1000/-  83.1/2000/- | 0.001–3.0 | [35] |
|  |  | -Robust organic-metal coordination framework, high electrochemical reversibility |  |  |  |
| **Aza-COF** | Condensation reaction using hexaketocyclohexane and 1,2,4,5-benzenetetramine | -Well-defined porosity and redox-active sites  -Embedding redox-active sites into a porous, crystalline π-conjugated framework | 523/60/1  241/3000/500 | 0.01–3.0 | [36] |
|  |  | -High theoretical capacity  -Phenazine-decorated channels  -Microporous structure with 2D sheets, π-conjugated framework |  |  |  |
| **Disodium terephthalate (Na_2_TP)** | Solvothermal method and acid-base reaction | -Utilization of PET waste to synthesize organic materials for Na-ion batteries  -Conversion of PET waste into terephthalic acid (TPA) and then into Na_2_TP | Na_2_TP-55%:  130.8/25.5/50  74.3/255/50  Na_2_TP/SWCNTs: 178.3/25.5/50  112.1/255/50 | 0.0–2.0 | [37] |
|  |  | -Eco-friendly, low-cost, modified Na_2_TP/SWCNTs anodes with enhanced specific capacity and stability |  |  |  |
| **Na-BDC** | Mechanochemical synthesis using shaker ball mill, sodium acetate and 1,4-benzenedicarboxylic acid (BDC) | -Fast, solvent-free synthesis, sustainable and scalable  -Deprotonation of carboxylate group facilitated by sodium acetate | 230/100/100 | 0.01–2.5 | [38] |
|  |  | -Environmentally friendly, reduced reaction time, solvent-free |  |  |  |
| **Trihydroxy-modulated Triazine-based Anode (TTHP-T)** | One-pot Friedel-Crafts reaction | -Simple structural modulation  -Trihydroxyphenyl functionalities conjugated with the triazine ring to enhance Na-ion trapping | 650/100/-  431/400/-  295/2000/-  497/100/1000 | 0.01–3.0 | [39] |
|  |  | -High battery capacity, stable cycling performance, good rate capability |  |  |  |

| **2,2′-bipyridine-4,4′-dicarboxylic acid disodium salt (Na-DCA)** | Mix 2,2′-bipyridine-4,4′-dicarboxylic acid and excess sodium hydroxide in ethanol, stir, filter, wash, and dry | -Investigates the substitution rule of N atoms in carboxylates  -Introduces N-doped reduced graphene oxide (NrGO) to improve conductivity and cycle stability. | 294/50/1  200/50/200  126/200/500 | 0.01–2.0 | [40] |
| --- | --- | --- | --- | --- | --- |
|  |  | -Forms stable resonance products  -Four-electron/Na^+^ redox mechanism,high specific capacity, stable SEI layer. |  |  |  |
| **[5, 10, 15, 20-tetrathienylporphinato] M (II) (MTTP, M=2H, Ni)**  **2,2′-bipyridine-4,4′-dicarboxylic acid disodium salt (Na-DCA)** | Thiophene-functionalized porphyrin molecules synthesized  Mix 2,2′-bipyridine-4,4′-dicarboxylic acid and excess sodium hydroxide in ethanol. | -Incorporation of thiophene functional groups and Ni(Ⅱ) ion into porphyrin molecules | NiTTP:  174/1000/1100  374/25/60  H_2_TTP:  134/1000/1000  203/25/60 | 0.01–3.0 | [41] |
|  |  | -Investigates the substitution rule of N atoms in carboxylates  -Introduces N-doped reduced graphene oxide (NrGO) to improve conductivity and cycle stability. |  |  |  |
| **HAT-PPDA** | HAT-6COOH and PPDA were subjected to a hydrothermal reaction in water at 200 °C for 72 hours. | -First report of a hexaazatriphenylene-based polymer as a universal anode for Li-/Na-/K-ion batteries.  -Utilizing HAT units with redox-active C═N and C═O bonds to enhance electron acceptance and storage capacity. | 348/100/50 | 0.01–3.0 | [42] |
|  |  | -High electron acceptance per unit (6 for Na^+^/K^+^, 9 for Li^+^), planar and conjugated structure, superior performance in multiple ion batteries. |  |  |  |
| **PMCDI** | Perylene anhydride, glycine, and imidazole were mixed and heated | -Introduction of amino acid groups  -Biomimetic structural modification by grafting amino acids onto PTCDA | 300/500/800 | 0.01–3.0 | [43] |
|  |  | -High stability and capacity due to hydrogen bonding and non-conjugated carboxylic acid groups as Na^+^ storage sites. |  |  |  |
| **HHTP-TABQ COF** | Tetramino-benzoquinone (TABQ) was synthesized and mixed with hexahydroxytriphenylene (HHTP) in the presence of TFA and NMP. | -Integration of C═N and C═O dual-active sites within a fully π-conjugated framework.  -Design of a 2D COF with abundant nitrogen heteroatoms and conjugated skeleton | 202/5000/1000  349/100/100  459/100/-  395/200/-  321/500/-  266/1000/-  213/2000/-  148/5000/- | 0.01–3.0 | [44] |
|  |  | -High specific surface area, and ordered pores  -π-Conjugated framework, and intrinsic insolubility. |  |  |  |
| **HATN-6OH** | synergetic coupling of catechol/ortho-quinone functional groups and HATN conjugated core structures | -Synergetic coupling of catechol/ortho-quinone and HATN conjugated core structures  -Integration of multiple redox-active sites and π–π interactions | 155/10000/3000  554/100/-  533/200/-  471/500/-  379/1000/-  320/2000/-  254/5000/-  202/10000/- | 0.01–3.0 | [45] |
|  |  | -High electron delocalization  -Strong intermolecular hydrogen bonding  -High ionic diffusivity, and excellent thermal stability. |  |  |  |
| **CityU-9** | Cross-coupling reaction between benzenehexathiol (BHT) and tetrafluoro-1,4-benzoquinone (TFBQ) | -Integration of benzoquinone and thioether motifs  -Sulfur induction for improved conductivity and rigid skeleton for intrinsic insolubility. | 588/200/600 | 0.01–3.0 | [46] |
|  |  | -High electron delocalization  -High ionic diffusivity, plentiful active sites  -Excellent thermal stability  -Limited solubility in electrolytes. |  |  |  |
| **CPTA** | TA:cyanothiophene reacted with trifluoromethanesulfonic acid.  CPTA: TA polymerized with FeCl_3_ in dichloroethane | -Conjugated and hierarchical structure with dual active sites  -Oxidative polymerization and structural engineering | 230/23/- | 0.01–3.0 | [47] |
|  |  | -High electron delocalization  -High ionic diffusivity, plentiful active sites  exceptional structural stability  -Limited solubility in electrolytes. |  |  |  |

| **TPPA-PI** | Two-step chemical imidization | -Incorporation of TPPA units with different bridge functionalizations  -Leveraging their structural advantages. | 218/50/50 | 0.02–2.0 | [48] |
| --- | --- | --- | --- | --- | --- |
|  |  | -Good specific capacity, improved cycling stability  -Versatile battery electrode applications |  |  |  |
| **Sodium 2-amino-4-thiazol-derived polymer (PATANa)** | ATA and APS were dissolved in DI water, then combined and refluxed. The resulting product, PATA, was neutralized with NaOH. | -Novel saline polymer anode for SIBs  -Salinization of polymer | 303/100/100  190/1000/1000  322/100/-  250/200/-  219/500/-  174/1000/-  164/2000/- | 0.01–3.0 | [49] |
|  |  | -2D nanosheet structure  -Larger contact area with electrolyte  -Shorter ion-migration path  -Improved ion-diffusion kinetics  -Insolubility in electrolyte  -Excellent cycling stability and rate capability |  |  |  |
| **PyIm-COF (Pyrenoimidazole-based COF)** | Self-condensation of 4,4′-(4,5,9,10-tetraoxo-4,5,9,10-tetrahydropyrene-2,7-diyl)dibenzaldehyde (TOTPA) | -Nitrogen-rich conjugated COF with imidazole linkages  -Extended Conjugated Backbone | 250/5000/2500 | 0.01–3.0 | [50] |
|  |  | -High capacity, excellent cycling stability  -High rate performance  -Nitrogen-rich active sites |  |  |  |
| **BDTTS (Tetrathiafulvalene Carboxylate)** | One-pot reaction of 4,4′,4′′,4′′′-([2,2′-bi(1,3-dithiolidene)]-4,4′,5,5′-tetrabenzoic acid (BDTTA) and sodium hydroxide in ethanol | -Tetrathiafulvalene with multiple active sites  -Rigid π-conjugated planar structure | 230/50/100  233/50/-  98/1250/- | 0.01–3.0 | [51] |
|  |  | -Low solubility in electrolyte  -High conductivity, multiple active sites |  |  |  |

**Table S4.** Impedance parameters of the D/A-CON-10-F electrode are fitted through the equivalent circuit model in **Figure 6i**.

|  | **D/A-CON-10-F** | | | | |
| --- | --- | --- | --- | --- | --- |
|  | **R_s_ (Ω)** | **R_f_ (Ω)** | **R_ct_ (Ω)** | **R_ion_ (Ω)** | **R’_ion_ (Ω)** |
| **Fresh cell** | 0.41 | - | 60.50 | - | - |
| **1^st^ discharge** | 0.49 | 28.70 | 5.20 | 5.40 | - |
| **1^st^ charge** | 1.41 | 4.70 | 31.90 | - | - |
| **2^nd^ discharge** | 0.73 | 31.20 | 6.40 | 6.30 | - |
| **2^nd^ charge** | 1.71 | 1.80 | 24.70 | - | - |
| **5^th^ discharge** | 0.42 | 30.50 | 5.30 | 4.70 | - |
| **5^th^ charge** | 1.91 | 2.60 | 19.50 | - | - |
| **50^th^ discharge** | 0.93 | 24.70 | 6.10 | 4.90 | - |
| **50^th^ charge** | 2.02 | 2.20 | 22.30 | - | - |
| **100^th^ discharge** | 0.76 | 10.20 | 5.80 | 4.60 | - |
| **100^th^ charge** | 1.81 | 3.10 | 23.10 | - | - |
| **300^th^ discharge** | 0.58 | 9.30 | 5.20 | 2.80 | - |
| **300^th^ charge** | 2.43 | 3.40 | 15.80 | - | - |

**Table S5.** Impedance parameters of the D/A-CON-10 electrode are fitted through the equivalent circuit model in **Figure 6i**.

|  | **D/A-CON-10** | | | | |
| --- | --- | --- | --- | --- | --- |
|  | **R_s_ (Ω)** | **R_f_ (Ω)** | **R_ct_ (Ω)** | **R_ion_ (Ω)** | **R’_ion_ (Ω)** |
| **Fresh cell** | 2.36 | 0.00 | 103.70 | - | - |
| **1^st^ discharge** | 0.67 | 33.10 | 6.90 | 6.40 | 7.90 |
| **1^st^ charge** | 2.48 | 10.60 | 70.20 | - | - |
| **2^nd^ discharge** | 1.16 | 66.40 | 16.50 | 18.20 | 21.80 |
| **2^nd^ charge** | 2.80 | 5.20 | 40.50 | - | - |
| **5^th^ discharge** | 3.24 | 60.70 | 16.60 | 16.80 | 22.70 |
| **5^th^ charge** | 2.83 | 3.30 | 44.10 | - | - |
| **50^th^ discharge** | 0.77 | 68.50 | 14.70 | 12.60 | 19.40 |
| **50^th^ charge** | 2.81 | 4.20 | 45.80 | - | - |
| **100^th^ discharge** | 1.50 | 62.20 | 13.90 | 13.70 | 22.00 |
| **100^th^ charge** | 3.05 | 5.10 | 37.20 | - | - |
| **300^th^ discharge** | 1.84 | 58.70 | 11.30 | 15.80 | 21.80 |
| **300^th^ charge** | 3.20 | 10.20 | 49.90 | - | - |

**Supplementary References**

[1] M.-S. Kim, M. Lee, M.-J. Kim, Y. K. Jeong, J. K. Park, S.-M. Paek, *J. Mater. Chem. A* **2020**, *8*, 17790–17799.

[2] Y. Zhang, Y. Tang, J. Deng, W. R. Leow, H. Xia, Z. Zhu, Z. Lv, J. Wei, W. Li, C. Persson, O. I. Malyi, M. Antonietti, X. Chen, *ACS Mater. Lett.* **2019**, *1*, 519–525.

[3] a) V. Augustyn, P. Simon, B. Dunn, *Energy Environ. Sci.* **2014**, *7*, 1597; b) C. Choi, D. S. Ashby, D. M. Butts, R. H. DeBlock, Q. Wei, J. Lau, B. Dunn, *Nat. Rev. Mater.* **2019**, *5*, 5–19.

[4] a) L. Hu, M. Jin, Z. Zhang, H. Chen, F. Boorboor Ajdari, J. Song, *Adv. Funct. Mater.* **2022**, *32*, 2111560; b) D. Pinheiro, M. Pineiro, J. S. S. de Melo, *Commun. Chem.* **2021**, *4*, 1–10; c) R. Wang, X. Chen, Z. Huang, J. Yang, F. Liu, M. Chu, T. Liu, C. Wang, W. Zhu, S. Li, S. Li, J. Zheng, J. Chen, L. He, L. Jin, F. Pan, Y. Xiao, *Nat. Commun.* **2021**, *12*, 1–10.

[5] T. H. Wan, M. Saccoccio, C. Chen, F. Ciucci, *Electrochim. Acta* **2015**, *184*, 483–499.

[6] M. Lee, S. Park, B. Bae, Y. K. Jeong, J.-M. Oh, J. K. Park, S.-M. Paek, *Chem. Eng. J.* **2023**, *477*, 147072

[7] M.-S. Kim, W.-J. Lee, S.-M. Paek, J. K. Park, *ACS Appl. Mater. Interfaces* **2018**, *10*, 32102–32111.

[8] M. Lee, M.-S. Kim, J.-M. Oh, J. K. Park, S.-M. Paek, *ChemSusChem* **2021**, *14*, 3244–3256.

[9] F. Tian, Y. Zhang, L. Liu, Y. Zhang, Q. Shi, Q. Zhao, Y. Cheng, C. Zhou, S. Yang, X. Song, *Langmuir* **2020**, *36*, 15075–15081.

[10] M.-M. Hu, H. Huang, Q. Gao, Y. Tang, Y. Luo, Y. Deng, L. Zhang, *Energy Fuels* **2021**, *35*, 1851–1858.

[11] S. Haldar, D. Kaleeswaran, D. Rase, K. Roy, S. Ogale, R. Vaidhyanathan, *Nanoscale Horiz* **2020**, *5*, 1264–1273.

[12] L.-Y. Wang, C. Ma, X. Wei, B. Chang, K.-X. Wang, J.-S. Chen, *J. Mater. Chem. A* **2020**, *8*, 8469–8475.

[13] C. Luo, J. J. Shea, J. Huang, *J. Power Sources* **2020**, *453*, 227904.

[14] C. Ma, L.-Y. Wang, M.-H. Shu, C.-C. Hou, K.-X. Wang, J.-S. Chen, *J. Mater. Chem. A* **2021**, *9*, 11530–11536.

[15] M. Zhang, Y. Tong, J. Xie, W. Huang, Q. Zhang, *Chemistry* **2021**, *27*, 16754–16759.

[16] J. Zhao, M. Zhou, J. Chen, L. Tao, Q. Zhang, Z. Li, S. Zhong, H. Fu, H. Wang, L. Wu, *Chem. Eng. J.* **2021**, *425*, 131630.

[17] K. Jia, L. Zhu, F. Wu, *ChemSusChem* **2021**, *14*, 3124–3130.

[18] J. Yan, X.-L. Chen, Y. Cui, G.-Z. Yang, Z.-L. Zheng, D.-S. Bin, D. Li, *ACS Appl. Mater. Interfaces* **2022**, *14*, 49641–49649.

[19] Y. Bo, W. Wu, R. Guo, M. Cao, Y. Liang, M. Wang, W. Yu, L. Zhang, J. Zhang, *Electrochim. Acta* **2022**, *405*, 139628.

[20] X. Yang, Y. Jin, B. Yu, L. Gong, W. Liu, X. Liu, X. Chen, K. Wang, J. Jiang, *Sci. China Chem.* **2022**, *65*, 1291–1298.

[21] K. Jia, H. Liu, G. Huang, J. Zhang, X. Liu, L. Li, L. Zhu, F. Wu, *J. Mater. Chem. A* **2022**, *10*, 14917–14922.

[22] S. Vedachalam, P. Sekar, C. Nithya, N. Murugesh, R. Karvembu, *ACS Appl. Energy Mater.* **2022**, *5*, 557–566.

[23] K. Jia, J. Zhang, P. Hu, L. Zhu, X. Li, X. Liu, R. He, F. Wu, *J. Colloid Interface Sci.* **2022**, *623*, 637–645.

[24] J. Huang, K. I. E. Callender, K. Qin, M. Girgis, M. Paige, Z. Yang, A. Z. Clayborne, C. Luo, *ACS Appl. Mater. Interfaces* **2022**, *14*, 40784–40792.

[25] S. Chae, T. Lee, W. Kwon, H. Kang, H. J. Seo, E. Kim, E. Jeong, J. H. Lee, S. G. Lee, *Chem. Eng. J.* **2023**, *453*, 139805.

[26] S. Wu, J. Yang, Y. Ni, Y. Han, W. Chen, J. Wu, *ACS Appl. Mater. Interfaces* **2024**, *16*, 39349–39355.

[27] Y. Liu, Z. Yao, P. Vanaphuti, X. Yang, L. Mei, X. Zhu, S. Liu, Y. Wang, *Cell Rep. Phys. Sci.* **2023**, *4*, 101240.

[28] S. Li, H. Wu, C. Wu, M. Jin, H. Yi, S.-Y. Lu, Y. Zhang, *J. Electroanal. Chem.* **2023**, *950*, 117852.

[29] J. Lee, Y. Kim, S. Park, K. H. Shin, G. Jang, M. J. Hwang, D. Kim, K.-A. Min, H. S. Park, B. Han, D. K. P. Ng, L. Y. S. Lee, *Energy Environ. Mater.* **2023**, *6*, e12468.

[30] M. K. Shehab, H. M. El-Kaderi, *ACS Appl. Mater. Interfaces* **2024**, *16*, 14750–14758.

[31] L. Zhao, S. Qin, F. Wu, L. Zhu, Q. Han, L. Xie, X. Qiu, H. Wei, L. Yi, X. Cao, *Chin. Chem. Lett.* **2024**, 110246.

[32] J. Tzadikov, N. R. Levy, L. Abisdris, R. Cohen, M. Weitman, I. Kaminker, A. Goldbourt, Y. Ein-Eli, M. Shalom, *Adv. Funct. Mater.* **2020**, *30*, 2000592.

[33]T. Yang, C. Zhang, W. Ma, X. Gao, C. Yan, F. Wang, J.-X. Jiang, *Solid State Ion.* **2020**, *347*, 115247.

[34]Q. Zhao, W. Zhao, C. Zhang, Y. Wu, Q. Yuan, A. K. Whittaker, X. S. Zhao, *Energy Fuels* **2020**, *34*, 5099–5105.

[35]T. Gu, S. Gao, J. Wang, S. Cao, K. Wang, M. Zhou, K. Jiang, *ChemElectroChem* **2020**, *7*, 3517–3521.

[36]M. K. Shehab, K. S. Weeraratne, T. Huang, K. U. Lao, H. M. El-Kaderi, *ACS Appl. Mater. Interfaces* **2021**, *13*, 15083–15091.

[37]L. Kumaresan, K. P. Kirubakaran, M. Priyadarshini, K. Kasiviswanathan, C. Senthil, C. W. Lee, K. Vediappan, *Sustain. Mater. Technol.* **2021**, *28*, e00247.

[38]D. N. Rainer, A. V. Desai, A. R. Armstrong, R. E. Morris, *J. Mater. Chem. A* **2021**, *9*, 27361–27369.

[39]Y. Shan, Y. He, Y. Gu, Y. Sun, N. Yang, H. Jiang, F. Wang, C. Li, D.-E. Jiang, H. Liu, X. Zhu, S. Dai, *Chem. Eng. J.* **2022**, *430*, 133055.

[40]K. Holguin, K. Qin, E. P. Kamphaus, F. Chen, L. Cheng, G.-L. Xu, K. Amine, C. Luo, *J. Power Sources* **2022**, *533*, 231383.

[41]J. Zhang, C. Ye, Y. Liao, C. Sun, Y. Zeng, J. Xiao, Z. Chen, W. Liu, X. Yang, P. Gao, *Mater. Futures* **2023**, *2*, 035101.

[42]J. Zou, K. Fan, X. Wang, Y. Chen, Y. Cao, H. Dai, C. Zhang, M. Fu, Y. Gao, H. Liu, C. Wang, *Chem. Eng. J.* **2023**, *460*, 141703.

[43]S. Zhang, X. Zhao, T. Li, J. Liu, F. Huang, T. Lin, *Sci. China Mater.* **2023**, *66*, 3817–3826.

[44]M. Zhang, Y. Tong, Z. Sun, J. Wang, Y. Lin, F. Kang, Q. Zhang, W. Huang, *Chem. Mater.* **2023**, *35*, 4873–4881.

[45]P. Yang, Z. Wu, S. Wang, M. Li, H. Chen, S. Qian, M. Zheng, Y. Wang, S. Li, J. Qiu, S. Zhang, *Angew. Chem.* **2023**, *62*, e202311460.

[46]S. Xu, C. Wang, T. Song, H. Yao, J. Yang, X. Wang, J. Zhu, C.-S. Lee, Q. Zhang, *Adv. Sci.* **2023**, *10*, DOI 10.1002/advs.202304497.

[47]M. K. Shehab, K. S. Weeraratne, O. M. El-Kadri, V. K. Yadavalli, H. M. El-Kaderi, *Macromol. Rapid Commun.* **2023**, *44*, 2200782.

[48]A. L. Lubis, F. Baskoro, T.-H. Lin, H. Q. Wong, G.-S. Liou, H.-J. Yen, *ACS Appl. Mater. Interfaces* **2023**, *16*, 48722–48735.

[49]H. Kang, Y. Pang, Q. Ma, R. Jin, J. Li, H. Li, L. Zhang, Y. Dong, J. Yue, C. Zhang, *Dalton Trans.* **2023**, *52*, 4760–4767.

[50]L. Liu, Y. Gong, Y. Tong, H. Tian, X. Wang, Y. Hu, S. Huang, W. Huang, S. Sharma, J. Cui, Y. Jin, W. Gong, W. Zhang, *CCS Chem* **2024**, *6*, 1255–1263.

[51]Y. Luo, K. Jia, X. Li, J. Zhang, G. Huang, C. Zhong, L. Zhu, F. Wu, *ChemSusChem* **2024**, *17*, e202301847.
